# The multifaceted roles of Arbuscular Mycorrhizal Fungi in peanut responses to salt, drought, and cold stress

**DOI:** 10.1186/s12870-023-04053-w

**Published:** 2023-01-16

**Authors:** Yuexu Liu, Jinhao Lu, Li Cui, Zhaohui Tang, Dunwei Ci, Xiaoxia Zou, Xiaojun Zhang, Xiaona Yu, Yuefu Wang, Tong Si

**Affiliations:** 1 Shandong Provincial Key Laboratory of Dryland Farming Technology,College of Agronomy, Qingdao Agricultural University, Qingdao, 266109 China; 2 Institute of Crop Germplasm Resources, Shandong Academy of Agricultural Sciences (SAAS), Jinan, 250100 China; 3 Shandong Peanut Research Institute, Qingdao, 266199 China

**Keywords:** AMF, Legumes, Environmental stress, Plant physiology, Metabolic pathway

## Abstract

**Background:**

Arbuscular Mycorrhizal Fungi (AMF) are beneficial microorganisms in soil-plant interactions; however, the underlying mechanisms regarding their roles in legumes environmental stress remain elusive. Present trials were undertaken to study the effect of AMF on the ameliorating of salt, drought, and cold stress in peanut (*Arachis hypogaea* L.) plants. A new product of AMF combined with *Rhizophagus irregularis* SA, *Rhizophagus clarus* BEG142, *Glomus lamellosum* ON393, and *Funneliformis mosseae* BEG95 (1: 1: 1: 1, w/w/w/w) was inoculated with peanut and the physiological and metabolomic responses of the AMF-inoculated and non-inoculated peanut plants to salt, drought, and cold stress were comprehensively characterized, respectively.

**Results:**

AMF-inoculated plants exhibited higher plant growth, leaf relative water content (RWC), net photosynthetic rate, maximal photochemical efficiency of photosystem II (PSII) (Fv/Fm), activities of antioxidant enzymes, and K^+^: Na^+^ ratio while lower leaf relative electrolyte conductivity (REC), concentration of malondialdehyde (MDA), and the accumulation of reactive oxygen species (ROS) under stressful conditions. Moreover, the structures of chloroplast thylakoids and mitochondria in AMF-inoculated plants were less damaged by these stresses. Non-targeted metabolomics indicated that AMF altered numerous pathways associated with organic acids and amino acid metabolisms in peanut roots under both normal-growth and stressful conditions, which were further improved by the osmolytes accumulation data.

**Conclusion:**

This study provides a promising AMF product and demonstrates that this AMF combination could enhance peanut salt, drought, and cold stress tolerance through improving plant growth, protecting photosystem, enhancing antioxidant system, and regulating osmotic adjustment.

**Supplementary Information:**

The online version contains supplementary material available at 10.1186/s12870-023-04053-w.

## Background

Agricultural crops are constantly exposed to a diversity of environmental stress worldwide including salt, drought, and cold throughout their sessile life cycles [1–3]. To survive from environmental stress, plants have intrinsically evolved sophisticated physiological and biochemical mechanisms to achieve tolerance in mitigating various internal and external stimuli [4, 5]. All environmental constraints result in an accumulation of reactive oxygen species (ROS) [6, 7]. ROS are known as a double-edge sword acting on the one hand allowing oxidative signalling that underpin plants adaptation to environmental stress and on the other as toxic by-products during stressful conditions [8–10]. It is therefore, plausible that maintaining a delicate balance between ROS production and scavenging is vital to plant survival, allowing plants better adaptation to the changing global climate, leading to a higher agricultural production.

Legume crops belong to family Fabaceae, which are excellent source of proteins, vitamins as well as fatty acids for both humans and animals [11–13]. It is well accepted that legume crops are among the most important crop species for ensuring global food security in the coming decades. With the publications of the IPCC 4^th^ and 5^th^ reports [14, 15]; however, global climate change is strongly influencing the behavior of legumes in resistance to abiotic stress and eventually reducing the crop production [16–18]. As an important leguminous crop, peanut (*Arachis hypogaea* L.) is globally cultivated in most tropical, subtropical, and temperate regions, which adapts to a wide range of climatic and soil conditions [19–21]. Peanut is relatively sensitive to salt and drought stress [22, 23]. Usually, improper irrigation and excessive evaporation of soil moisture result in aggravated soil salinization, induces damage to peanut crops [24, 25]. Under the changing climate, the frequent and unpredictable drought and cold periods occur during the life cycle of peanut, and the seed yields are negatively impacted [26, 27]. In this scenario, special land management practices to be adopted in peanut production are ongoing endeavors for coping with unprecedented changes under global environmental change.

Arbuscular mycorrhizal fungi (AMF) symbiotically associate with most terrestrial crops which occur extensively in soils with bio-amelioration performances. It is reported that AMF alleviate crop abiotic stress by modifying good root morphology through the extra-radical mycelium [28, 29] as well as improving the absorption of water and nutrients in the soil [30, 31]. Moreover, AMF inoculation increased the host plants’ photosynthesis rate by enhancing the photosynthesis ability [32, 33] and declined the generation of toxic radicals by maintaining higher enzyme activities [30, 34]. Several studies also revealed that inoculation of AMF improved crop tolerance to environmental stress through regulating the rhizosphere microbial community and simulating osmolytes in their host plants [35, 36]. Despite extensive research, however, the detailed physiological and metabolomic mechanisms underlying multiple environmental stress response of AMF in legumes have not been firmly established.

The nodulation of rhizobia has long been regarded as an effect approach in leguminous crop production, which plays crucial roles in biological nitrogen fixation, plant growth, and plant environmental stress resistance [22, 37, 38]. To date, few reports in the literature have investigated the inter-specific interactions between AMF, rhizobia, and leguminous crop species, especially under environmental stressful conditions. Thus, the positive roles of AMF inoculation might be counteracted as the result of the inter-specific competition underground. Furthermore, recent researches on the alleviation effects of AMF have mainly focused on individual AMF inoculation [39–41], with little attention on the effects of the combination of AMF species in multiple environmental stress responses [42, 43]. Therefore, it is plausible that inoculation with a combination of AMF species could represent an economic, competitive, and eco-friendly choice, for the purpose of increasing the growth and production of peanut under environmental constraints.

To better address the impact of AMF on the responses of peanut to abiotic stress, the present investigation was carried out to determine the multifaceted roles of a new combination of AMF species *Rhizophagus irregularis* SA, *Rhizophagus clarus* BEG142, *Glomus lamellosum* ON393, and *Funneliformis mosseae* BEG95 (1: 1: 1: 1, w/w/w/w) on environmental stress tolerance in peanut plants. Pot-grown experiments were performed by inoculating the roots with/without AMF and the responses of peanut plants to salt, drought, and cold stress were comprehensively characterized, respectively. This study explored crucial physiological and metabolomic mechanisms regarding AMF-mediated peanut environmental stress responses, and the related results might have potential implication for achieving legumes security and ensuring sustainable agriculture under global climate change.

## Materials

### Plant and fungal materials

Peanut variety Huayu 25 (abbreviated as “HY25”) obtained from Shandong Peanut Research Institute (Qingdao, Shandong Province, China) was utilized as the experimental material in the present study, which is also a major-cultivated peanut variety in Shandong Province.

The AMF inoculum used in this study was a mixture of four AMF species (1: 1: 1: 1, w/w/w/w): *Rhizophagus irregularis* SA (~40 spores/g inoculum), *Rhizophagus clarus* BEG142 (~40 spores/g inoculum), *Glomus lamellosum* ON393 (~36 spores/g inoculum), and *Funneliformis mosseae* BEG95 (~45 spores/g inoculum). The detailed AMF information can be found on the International Bank for the Glomeromycota (http://www.i-beg.eu/) and the International Collection of (Vesicular) Arbuscular Mycorrhizal Fungi (https://invam.ku.edu/).

In the past two years, our preliminary screening experiment demonstrated that this AMF combination exhibited better effects in peanut growth and stress resistance than other kinds of combinations or individual AMF application (data not shown). The inoculum was prepared by cultivation with maize (*Zea mays* L.) as the host plant in a mixture of zeolite and sand (1:1, w/w) and has been reproduced for two consecutive years. The prepared inoculum for the present study consisted of spores (~40 spores/g inoculum), extraradical hyphae, and macerated maize roots. All of the AMF species were provided by Masaryk University (Brno, Czech Republic).

### Experimental design

A pot-grown experiment was conducted at the College of Agronomy, Qingdao Agricultural University, Qingdao, Shandong Province, China from May to August, 2021. The seeds of uniform sizes were firstly soaked in 2% (v/v) sodium hypochlorite solution for approximately 10 min and rinsed for two times in sterile distilled water. The seeds were then soaked in distilled water under dark at 25℃ for 24 h and transferred to wet filter paper under dark at 25℃ for another 24 h prior to planting. Afterward each disinfected polystyrene pot (inner diameter of 9 cm, depth of 8 cm, and with one small hole at the bottom) was filled with 120 g of soil which was twice heat-sterilized at 121℃ for 1 h to remove the propagules present in the soil. The physical and chemical properties of the soil were as follows: pH of 6.74; bulk density of 1.21 g/cm^3^; organic matter of 24.4 g/kg; nitrogen of 90.2 mg/kg; phosphorous of 32.5 mg/kg; and potassium of 61.7 mg/kg. Then 0.2 g of AMF inoculum used for inoculation was applied into the soil at 3 cm below the soil level in each pot and one seed was planted into one pot at 2 cm below the soil level (referred to as “AMF”). Meanwhile, pots without AMF inoculation were added 0.2 g of autoclaved inoculum (referred to as “non-AMF”).

Then the pots were randomly arranged and kept in a greenhouse with the following climate conditions: photoperiod of 16/8 h (light/dark); photosynthetic photon flux density (PPFD) of 1,200 μmol m^-2^ s^-1^; air temperature of 25/18℃ (day/night); and air humidity of 70%. Each pot was well watered with 100 mL of distilled water at 6:00 p.m. (local time in China) with a 2-day interval. At 40 days after emergence, plants with uniform sizes were selected for the experiments. (1) For salt stress treatment, respective pots were well watered with 100 mL of 200 mM NaCl solution (NaCl treated) or distilled water (non-NaCl treated) thrice with a 2-day interval. The concentration of NaCl adopted in this study was based on our preliminary researches [19, 44, 45]. Finally, the average soil salt content of NaCl-treated and non-NaCl-treated pots were 1.89 and 0.56 g/kg, respectively. (2) For drought stress treatment, drought stress was imposed by withholding irrigation for 7 consecutive days and the well-watered plants were taken as control. Finally, the average soil water content of drought-treated and non-drought-treated pots were 34.09 and 88.56%, respectively. (3) For cold stress treatment, the plants were transferred to a climate chamber with the growth air temperature of 14/8℃ (day/night) for 7 consecutive days and the control plants were maintained at 25/18℃ (day/night). The other climate conditions remain unchanged as described above. Therefore the detailed 8 treatments included: (1) “non-AMF” (non-AMF + non-stress); (2) “AMF” (AMF inoculation + non-stress); (3) “NaCl” (non-AMF + salt stress); (4) “AMF+NaCl” (AMF inoculation + salt stress); (5) “Drought” (non-AMF + drought stress); (6) “AMF+Drought” (AMF inoculation + drought stress); (7) “Cold” (non-AMF + cold stress); (8) “AMF+Cold” (AMF inoculation + cold stress). For all of the treatments, the measurements were conducted at 7 days after the onset of the stress treatment and the samples were also taken simultaneously (Fig. S1). The average root AMF colonization in “AMF”, “AMF+NaCl”, “AMF+Drought”, and “AMF+Cold” was 26.3, 21.4, 19.8, and 25.5%, respectively. The third fully expanded leaves from the top of the main stem and the entire roots of the plants were measured and/or sampled unless otherwise stated. The experiment was performed with a randomized complete block design with three biological replicates for each treatment. Twenty-four similar-looking plants were used for each treatment.

### Measurements and data collection

#### AMF colonization and plant morphology analysis

Plant height was defined as the length from cotyledonary node to the growing point of the main stem. For the determination of shoot biomass, the shoots of the plants were separated, immediately washed twice with distilled water, followed by oven-dried at 105℃ for 30 min to deactivate all the enzymes, and then stove heated at 75℃ for at least 48 h before recording the constant dry weights. A total of 8 plants were analyzed for each replicate.

The root morphology was determined based on the method as described previously [44]. In brief, the dissected fresh roots were immediately washed twice with distilled water and scanned with a dual-lens scanning system (V700, SEIKO EPSON CORP., Japan). Then the obtained data including root volume, root length, root average diameter, and root surface area were analyzed using the WinRHIZO software (version 2013e, Regent Instruments Inc., Canada).

The method of trypan blue (0.05%, v/v) staining was taken to determine the AMF colonization of peanut roots as originally described [46]. After staining, the roots were observed under a microscope (DM6000M, Leica Co., Ltd., Wetzlar, Germany) and then the root AMF colonization (%) was calculated as no. of root segments (colonized)/no. of root segments (observed) × 100. The data of AMF colonization was collected in all treatments together with the determination of physiological parameters.

#### Gas exchange, chlorophyll fluorescence, and chlorophyll content

A portable photosynthesis system (Li-COR 6800, Lincoln, NE, USA) was utilized to measure the gas exchange data. The net photosynthetic rate (Pn), stomatal conductance (Gs), intercellular CO_2_ concentration (Ci), and transpiration rate (Tr) of the third fully expanded leaf of each plant were measured between 9:00-11:00 a.m. (local time in China) under the following conditions in the leaf chamber: air temperature of 25℃, PPFD of 1,200 μmol m^-2^ s^-1^, CO_2_ concentration of 400 μmol mol^-1^, and relative air humidity of 70%.

To determine the state of photosystem II (PSII), chlorophyll fluorescence was measured using an imaging pulse amplitude modulated (PAM) fluorimeter (IMAG-MAXI; Heinz Walz, Effeltrich, Germany) [45]. Before each measurement, leaves were dark-adapted for 30 min. The minimal fluorescence emission signal (Fo) was measured under a weak pulse of modulating light over 0.8 s, and maximal fluorescence (Fm) was induced by a saturating pulse of light (4,000 μmol m^-2^ s^-1^) applied over 0.8 s, after which the steady-state fluorescence yield (Fs) and light-adapted maximum fluorescence (Fm’) were recorded. Then, the maximal photochemical efficiency of PSII (Fv/Fm), the quantum yield of PSII photochemistry (ΦPSII), and photochemical quenching coefficient (qP) were calculated according to the formulas as described previously [47, 48]. All of the samples were analyzed with the same leaf as area of interest.

For the determination of the total chlorophyll content, 0.1 g of fresh leaf sample was excised immediately and extracted in 25 mL of anhydrous ethanol and acetone (1:1, v/v) solution and incubated for 12 h under dark at 4℃ (to reduce respiration). Then, the supernatant was mixed thoroughly and determined using an UV-Vis spectrophotometer (Cary 60, Agilent, USA) at both 647 and 663 nm. Then the total chlorophyll content was calculated according to HK Lichtenthaler and AR Wellburn [49].

#### Electron microscopy

The cytochemical observation of the leaves was conducted as described previously [50, 51]. In brief, the excised leaf tissue pieces (1-2 mm^2^) from differently treated leaves were fixed for 4 h at 4℃ in 1.25% (v/v) glutaraldehyde buffer and paraformaldehyde buffer (50 mM sodium cacodylate, pH 6.9) for 4 h. After dehydrating in a graded ethanol series (30-100%; v/v), the tissues were polymerized at 60℃ for 48 h. The sections were then cut to 70-90 nm with a Reichert-Ultracut E ultramicrotome before mounting on uncoated copper grids (300 mesh). Ultimately the ultrathin sections were observed with a transmission electron microscope (HT7700; Hitachi, Tokyo, Japan) operating at an accelerating voltage of 75 kV.

#### Histochemical staining and quantitative assay of H_2_O_2_ and O_2_^-.^

The *in situ* staining of the leaf hydrogen peroxide (H_2_O_2_) was carried out by using 3, 3-diaminobenzidine (DAB) solution (1 mg mL^-1^, pH 3.8), as previously described by H Thordal-Christensen, Z Zhang, Y Wei and DB Collinge [52] with minor modifications. Briefly, the excised leaves were immediately submerged in DAB solution and incubated for 12 h with a PPFD of 1,200 μmol m^-2^ s^-1^ at 25℃. Then leaves were decolorized in boiling ethanol (95%, v/v) for approximately 20 min until the brown spots were clearly visualized. After cooling, the leaves were transferred to lactic acid/phenol/water solution (1:1:1, v/v/v) and photographed immediately. For the *in situ* staining of superoxide anion (O_2_^−.^), the excised leaves were soaked in nitro blue tetrazolium (NBT) solution (1 mg mL^-1^, pH 6.1). Then, the leaves were incubated at 25℃ under dark for 6 h before bleaching in boiling ethanol (95%, v/v). The leaves were then transferred to lactic acid/phenol/water solution (1:1:1, v/v/v) and photographed.

For the quantitative assay of ROS, the leaf samples were excised, frozen in liquid nitrogen immediately, and stored at -80℃ prior to determination. The concentration of leaf H_2_O_2_ was measured based on the titanium peroxide complex absorbance change at 412 nm with minor modifications [53]. A standard concentration curve of H_2_O_2_ was performed for the data quantification. Meanwhile, the O_2_^−.^ production rate was quantified by monitoring the nitrite formation from hydroxylamine in the presence of O_2_^−.^ at 530 nm as previously described [54].

#### Relative water content, relative electrolyte conductivity, and lipid peroxidation determination

The relative water content (RWC) was measured following the method of Jensen et al. (2000) [55]. Firstly, the leaf samples were excised and fresh weight (FW) was recorded. Then the leaf samples were soaked in 10 mL of deionized water under dark for 4 h at 4℃ and turgid weight (TW) was recorded. Finally, the leaves were oven-dried at 75℃ for at least 48 h until constant dry weight (DW) was recorded. RWC was calculated with the following formula: RWC (%) = [(FW−DW) / (TW−DW)] × 100.

The relative electrolyte conductivity (REC) was determined to assess the cell membrane integrity as described by M Griffith and HCH Mclntyre [56]. In brief, the excised leaf samples were washed twice with deionized water, and then soaked in 10 mL of deionized water at 25℃. After 12 h, the conductivity (C1) was recorded using a conductivity bridge (DDS-307A, LEX Instruments Co., Ltd., China). Then, the solution was boiled for 30 min and the conductivity (C2) was determined again after cooling. REC was calculated with the following formula: REC (%) = C1/C2 × 100%.

The concentration of Malondialdehyde (MDA) was measured using 2-thiobarbituric acid (TBA) reaction for the estimation of cell lipid peroxidation. Briefly, 0.5 g of leaf sample was homogenized in 50 mM potassium phosphate buffer (pH 7.8) in an ice bucket and centrifuged at 12,000 *g* for 20 min. Then 4 mL 0.65% TBA in 20% trichloroacetic acid solution was added and the mixture was incubated at 95℃ for 20 min. After centrifuging at 4,000 *g* for 10 min, the absorbance of the supernatant was recorded at 440, 532, and 600 nm using an UV-Vis spectrophotometer (Cary 60, Agilent, USA). Then the concentration of MDA was calculated following the method of Hodges et al. (1999) [57].

#### Extraction and activity assays of antioxidant enzymes

Frozen leaf tissues (0.5 g) were homogenized with 5 mL ice-cold 50 mM phosphate buffer (pH 7.8) containing 0.2 mM EDTA, 20% (v/v) glycerol, 5 mM MgCl_2_, 1 mM dithiothreitol, and 2% poly vinyl pyrrolidone (w/v) using pre-chilled mortar and pestle. The homogenates were centrifuged at 12,000 *g* for 20 min at 4℃, and the resulting supernatants were collected for the determination of enzymatic activity. The total protein contents were first determined using the Coomassie Brilliant Blue reaction at 595 nm as originally described by MM Bradford [58]. Superoxide dismutase (SOD) activity was determined based on its ability to inhibit the photochemical reduction of NBT at 560 nm following the protocol of RRC Stewart and JD Bewley [59]. Guaiacol peroxidase (G-POD) activity was assayed against guaiacol substrate at 470 nm according to the method of I Cakmak and H Marschner [60] with minor modifications. Catalase (CAT) activity was analyzed based on H_2_O_2_ oxidation and measured as a decline at 240 nm according to HK Patra, M Kar and D Mishra [61]. Ascorbate peroxidase (APX) activity was assessed based on the oxidation of ascorbate at 290 nm as modified by Y Nakano and K Asada [62].

#### Measurements of sodium and potassium content

The measurements of sodium (Na^+^) and potassium (K^+^) contents were performed using the ion content determination method as previously described by Zhang et al. (2011) [63] with minor modifications. One hundred milligram of the leaf and root dry samples were powdered and immersed overnight in 5 mL concentrated sulfuric acid (30% H_2_O_2_ was taken as catalyst) to ensure that the samples were completely digested. Then the solution was transferred to a 50-mLvolumetric flask to a constant volume and diluted. Finally, the contents of Na^+^ and K^+^ were analyzed with an atomic absorption spectrophotometer (TAS-990, Purkinje General Instrument Co., Ltd.) and calculated as previously described [64].

#### Accumulation of total soluble sugar, sucrose, and free amino acids

The samples were firstly oven-dried at 105℃ for 30 min, followed by 75℃ for 3 days. Then, the samples were powdered in a high-speed ball mill (MM400; Retsch GmbH, Haan, Germany) and mixed thoroughly. The powder (0.1 g) was then extracted with 8 mL of 80% (v/v) ethanol in a 10-mL plastic tube at 80℃, after which the supernatant was collected after centrifugation at 3000 *g* for 30 min. The extraction was repeated twice and the collected extracts were combined followed by adding the same ethanol to the glass tube to a final volume of 25 mL. Then the extract was utilized to determine the contents of total soluble sugars, sucrose, and free amino acids after mixing thoroughly. The content of total soluble sugar was assessed using the anthrone method and the absorbance at 620 nm was recorded following the method of JAN Buysse and R Merckx [65]. The sucrose content was measured following the resorcinol method at 480 nm as originally described [65]. The content of free amino acids was further analyzed by the ninhydrin reaction at 570 nm following the method of S Moore and WH Stein [66] with minor modifications.

#### Non-targeted metabolites extraction and analysis

The entire root samples were collected according to the protocol of De Vos et al. (2007) [67]. The roots were excised and immediately washed thoroughly with 10 mM PBS buffer to remove the rhizosphere soil. Then the samples were frozen in liquid nitrogen for 15 min and stored in -80℃ before delivering to BioTree Biotechnology Co., Ltd. (Shanghai, China) for metabonomic analysis following the method of Theodoridis et al. (2008) [68]. Briefly, the metabolites were extracted for the UHPLC-QE-MS analysis. LC-MS/MS analyses were performed with an UHPLC system (Vanquish, Thermo Fisher Scientific) using a UPLC HSS T3 column (2.1 mm × 100 mm, 1.8 μm) coupled to Q Exactive HFX mass spectrometer (Orbitrap MS, Thermo). The raw data were then converted to the mzXML format using ProteoWizard and processed with an in-house program, which was developed using R and based on XCMS, for peak detection, extraction, alignment, and integration. The high resolution MS data were processed by MAPS software and identified by MS2 database. In order to visualize group separation and find significantly changed metabolites, supervised orthogonal projections to latent structures-discriminate analysis (OPLS-DA) was applied. Variance was analyzed in the data with variable importance in the projection (VIP) greater than 1 and *p*-value less than 0.05 as statistically significance. In addition, commercial databases including KEGG [69, 70] (http://www.genome.jp/kegg/) and MetaboAnalyst (http://www.metaboanalyst.ca/) were used for pathway enrichment analysis. In the present work, the positive and negative ionization modes have been analyzed separately while the combination results have been shown.

#### Statistical analysis

One-way analysis of variance (ANOVA) was performed using a SPSS statistical software (version 22.0, SPSS Inc., Chicago, IL, USA). The difference was considered to be statistically significant using Tukey’s test when *P* < 0.05.

## Results

### Inoculation of AMF promotes peanut environmental tolerance by improving plant growth

**Fig. 1:**
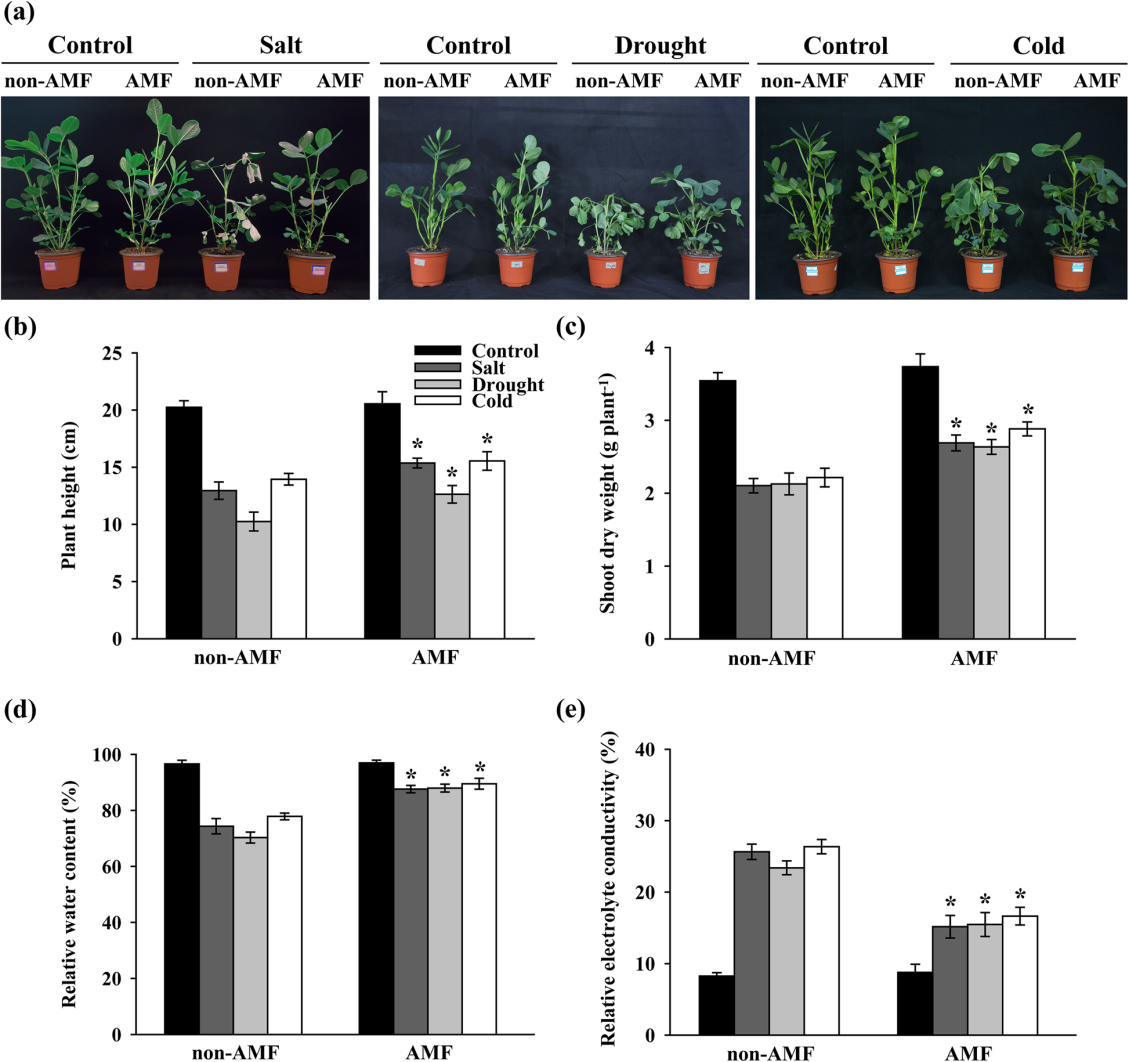
Effect of AMF on (**a**) plant growth, (**b**) plant height, (**c**) shoot dry weight, (**d**) relative water content (RWC), and (**e**) relative electrolyte conductivity (REC) in peanut plants under stressful conditions. The peanut seeds were inoculated with/without AMF before exposing to normal/salt/drought/cold growth conditions. The measurements were conducted on the 8^th^ day after the onset of stress treatments. Bars represent the mean values of three biological replicates with standard deviation; asterisks indicate a significant difference in comparison to non-AMF according to Tukey's test (*P* < 0.05)

Our first objective was to investigate the roles of AMF on peanut alleviation to environmental stress. The results indicated that peanut plants inoculated with AMF showed a resistance to all three types of stress at 7 days after the onset of the treatments (Fig 1a). Salt, drought and cold stress significantly decreased the plant height by 36.05, 49.38, and 31.11%, respectively compared to control. AMF did not show significant increase in plant height compared to control under normal growth conditions; however, AMF significantly increased plant height by 18.66, 23.25, and 11.47%, respectively compared to control under salt, drought, and cold stress (Fig 1b). Exposure of peanut plants to salt, drought, and cold significantly decreased the shoot dry weight by 40.64, 39.97, and 37.51%, respectively compared to control. Strikingly, inoculation with AMF resulted in a significant increase of shoot dry weight by 27.89, 23.90, and 30.17%, respectively compared with non-inoculated control (Fig. 1c). Salt, drought, and cold stress significantly reduced the RWC by 23.02, 27.21, and 19.39% compared with control. In contrast, plants treated with AMF significantly increased the RWC by 17.82, 25.09, and 14.96% respectively under salt, drought, and cold stress (Fig 1d). Similarly, salt, drought, and cold-induced inductions in REC were significantly reduced by 40.88, 33.89, and 36.86%, respectively, in AMF-treated plants (Fig. 1e), pointing out that AMF could be beneficial to maintain the integrity of plasma membrane of peanut leaves.

We then determined the role of AMF in the root morphology of peanut plants exposed to environmental stress. Salt, drought, and cold stress significantly reduced the root volume by 24.63, 22.94, and 20.92%, respectively compared to control. Notably, inoculation with AMF significantly induced the root volume by 28.76, 19.45, and 25.56%, respectively under salt, drought, and cold conditions (Fig. S2a & b). Additionally, salt, drought, and cold significantly reduced the total root length by 22.64, 21.46, and 18.31%, respectively whereas treatment with AMF significantly induced the total root length by 27.98, 19.83, and 21.97%, respectively under stressful conditions (Fig. S2c). Meanwhile, no significant changes in root average diameter were observed under any treatments (Fig. S2d). Moreover, compared with control of salt, drought, and cold treatment, AMF significantly increased the root surface area by 16.40, 12.94, and 18.47%, respectively under stressful conditions (Fig. S2e).

### Effects of AMF on photosynthesis, chlorophyll fluorescence, and chlorophyll content under environmental stress

**Fig. 2:**
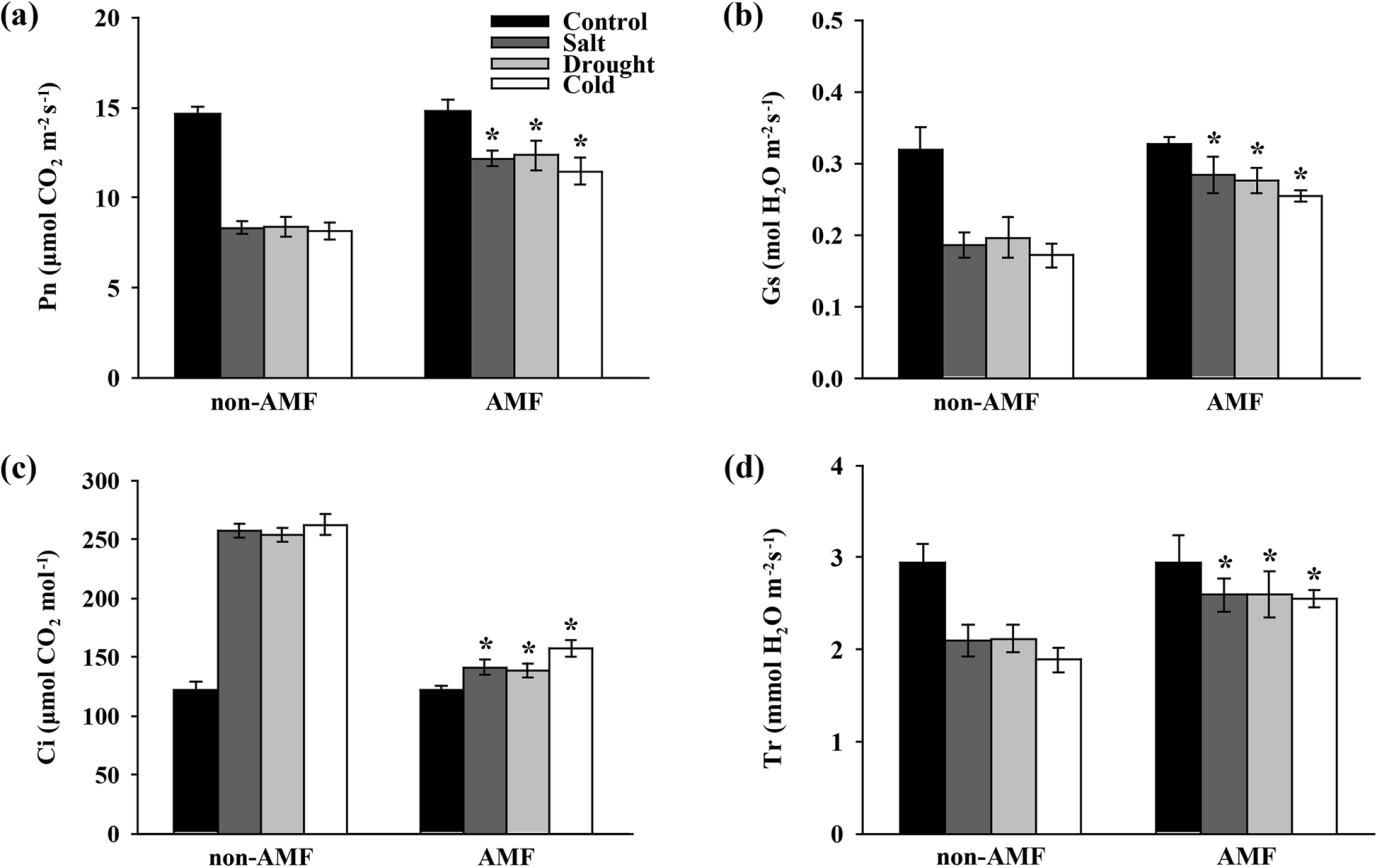
Effect of AMF on gas exchange in the third fully expanded leaves of peanut plants under stressful conditions. (**a**) Net photosynthetic rate (Pn), (**b**) stomatal conductance (Gs), (**c**) intercellular CO_2_ concentration (Ci), and (**d**) transpiration rate (Tr). The peanut seeds were inoculated with/without AMF before exposing to normal/salt/drought/cold growth conditions. The gas exchange parameters were measured on the 8^th^ day after the onset of stress treatments. Bars represent the mean values of three biological replicates with standard deviation; asterisks indicate a significant difference in comparison to non-AMF according to Tukey's test (*P* < 0.05)

Exposure to salt, drought, and cold stress significantly decreased the Pn by 43.03, 42.72, and 44.61%, respectively compared with control; however, inoculation with AMF significantly increased the Pn by 45.89, 47.37, and 41.22%, respectively (Fig. 2a). AMF-treated plants significantly increased the salt, drought, and cold stress-induced decreases in Gs by 52.46, 40.76, and 48.75%, respectively (Fig. 2b). Salt, drought, and cold stress showed 1.10, 1.07, and 1.15-fold increases in Ci, respectively, whereas inoculation with AMF significantly decreased the Ci by 45.10, 45.28, and 39.94%, respectively, in the presence of environmental stress (Fig. 2c). Tr was substantially decreased by 28.70, 27.91, and 35.75%, respectively, under salt, drought, and cold conditions whereas AMF significantly induced the Tr by 23.70, 22.45, and 34.97%, respectively (Fig. 2d).

**Fig. 3:**
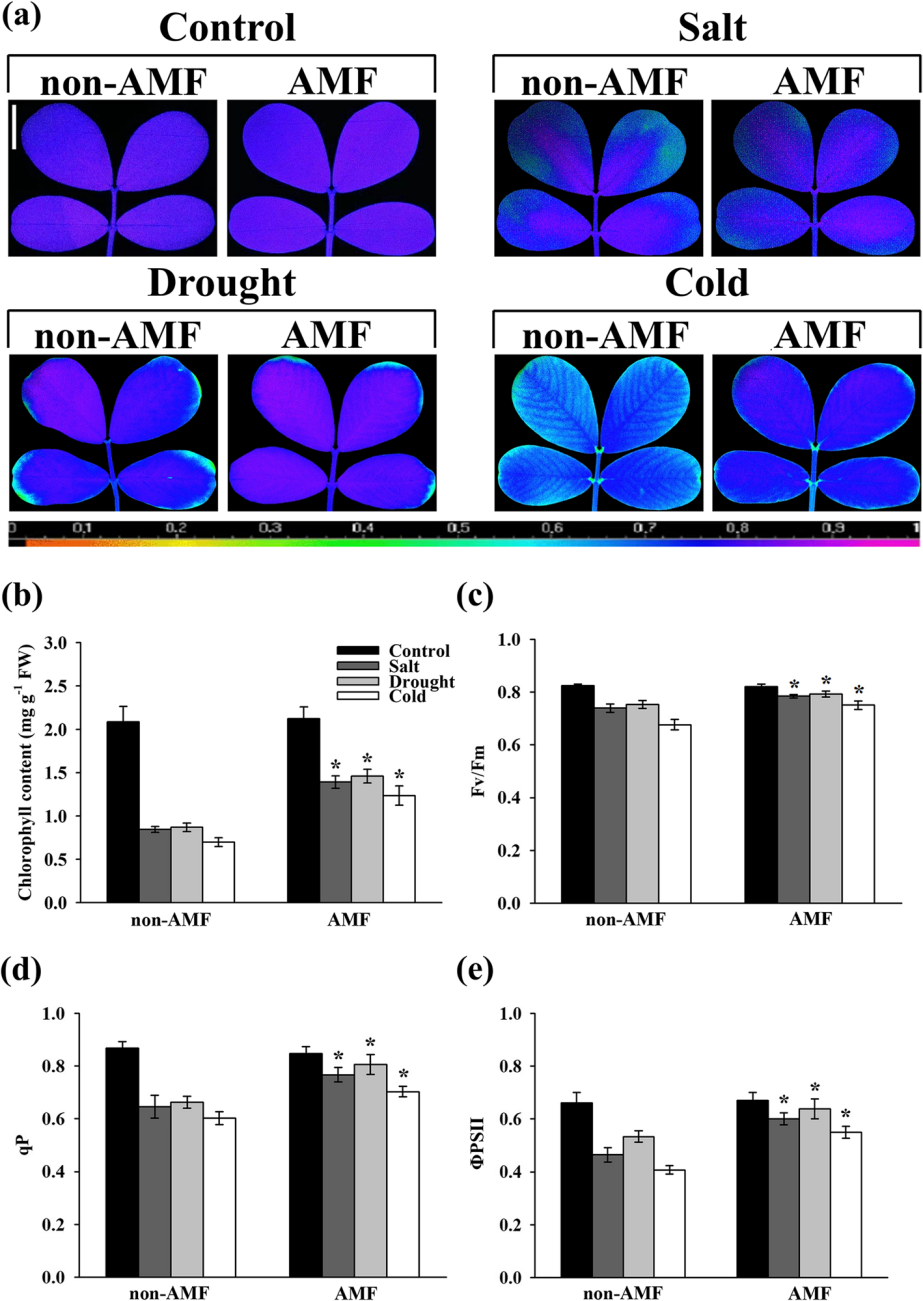
Effect of AMF on chlorophyll fluorescence and chlorophyll content in the third fully expanded leaves of peanut plants under stressful conditions. The peanut seeds were inoculated with/without AMF before exposing to normal/salt/drought/cold growth conditions. On 8^th^ day after the onset of stress treatments, the images showing (**a**) the maximal photochemical efficiency of photosystem II (PSII) (Fv/Fm) were taken. The false color code depicted at the bottom of the image ranges from 0 (black) to 1 (purple). Vertical bar = 1 cm. Meanwhile, the value of (**b**) the total chlorophyll content, (**c**) Fv/Fm, (**d**) photochemical quenching coefficient (qP), and (**e**) the quantum yield of PSII photochemistry (ΦPSII) were determined. Bars represent the mean values of three biological replicates with standard deviation; asterisks indicate a significant difference in comparison to non-AMF according to Tukey's test (*P* < 0.05)

The maximal photochemical efficiency of photosystem II (PSII) (Fv/Fm) was reduced by growth in the presence of salt, drought, and cold conditions, whereas the environmental stress-induced reduction in Fv/Fm was significantly lower in AMF-treated plants than in AMF non-treated plants (Fig. 3a & c). Consistent with Fv/Fm, salt, drought, and cold significantly reduced the chlorophyll content by 59.46, 58.30, and 66.52%, respectively compared with control. The chlorophyll content was significantly increased by 64.65, 67.77, and 76.94%, respectively in AMF-treated plants under stressful conditions (Fig. 3b). Similarly, AMF significantly increased salt, drought, and cold-reduced photochemical quenching coefficient (qP) by 18.72, 21.63, and 16.70%, respectively (Fig. 3d). In addition, the quantum efficiency of PSII photochemistry (ΦPSII) was substantially increased by 29.18, 19.66 and 35.05%, respectively in AMF-treated plants under salt, drought, and cold stress (Fig. 3e).

### Inoculation of AMF maintains intact thylakoids and mitochondria under stressful conditions

**Fig. 4:**
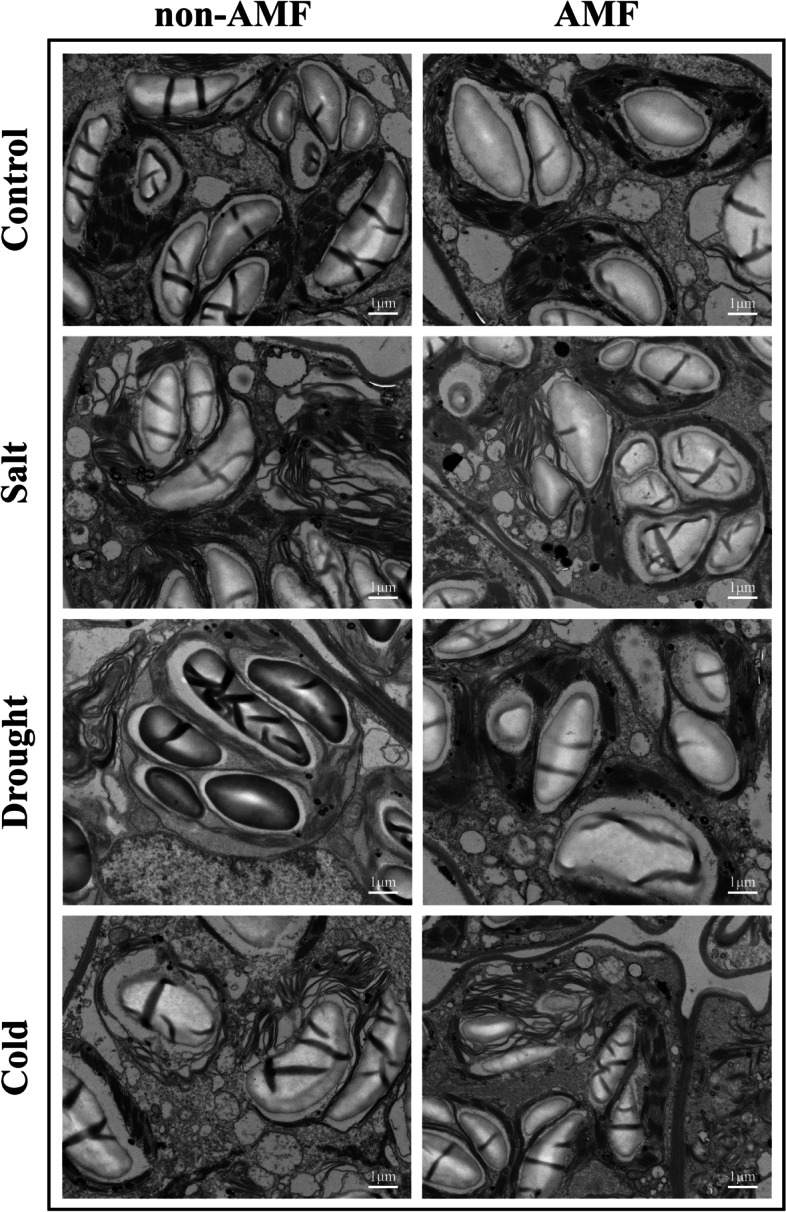
Effect of AMF on the integrity of thylakoids and mitochondria in the third fully expanded leaves of peanut plants under stressful conditions. The peanut seeds were inoculated with/without AMF before exposing to normal/salt/drought/cold growth conditions. On the 8^th^ day after the onset of stress treatments, the leaves were collected for cytochemical staining and observed with a transmission electron microscopy

Cytochemical staining method was utilized to further explore the protective role of AMF in peanut photosystem and mitochondrial respiratory chain. Electron micrographs showed that salt, drought, and cold stress induced varying degrees of chloroplast thylakoids destacking compared with control. By contrast, relatively intact thylakoids were detected in AMF-treated peanut plants under salt, drought, and cold stress. Similarly, exposure to salt, drought, and cold stress severely damaged the mitochondria ridges in mitochondria with hollow inside. Notably, the damages of mitochondria ultrastructure were significantly mitigated when the peanuts were inoculated with AMF (Fig. 4).

### Inoculation of AMF alleviates stress-induced oxidative damage and maintains higher antioxidants activities

**Fig. 5:**
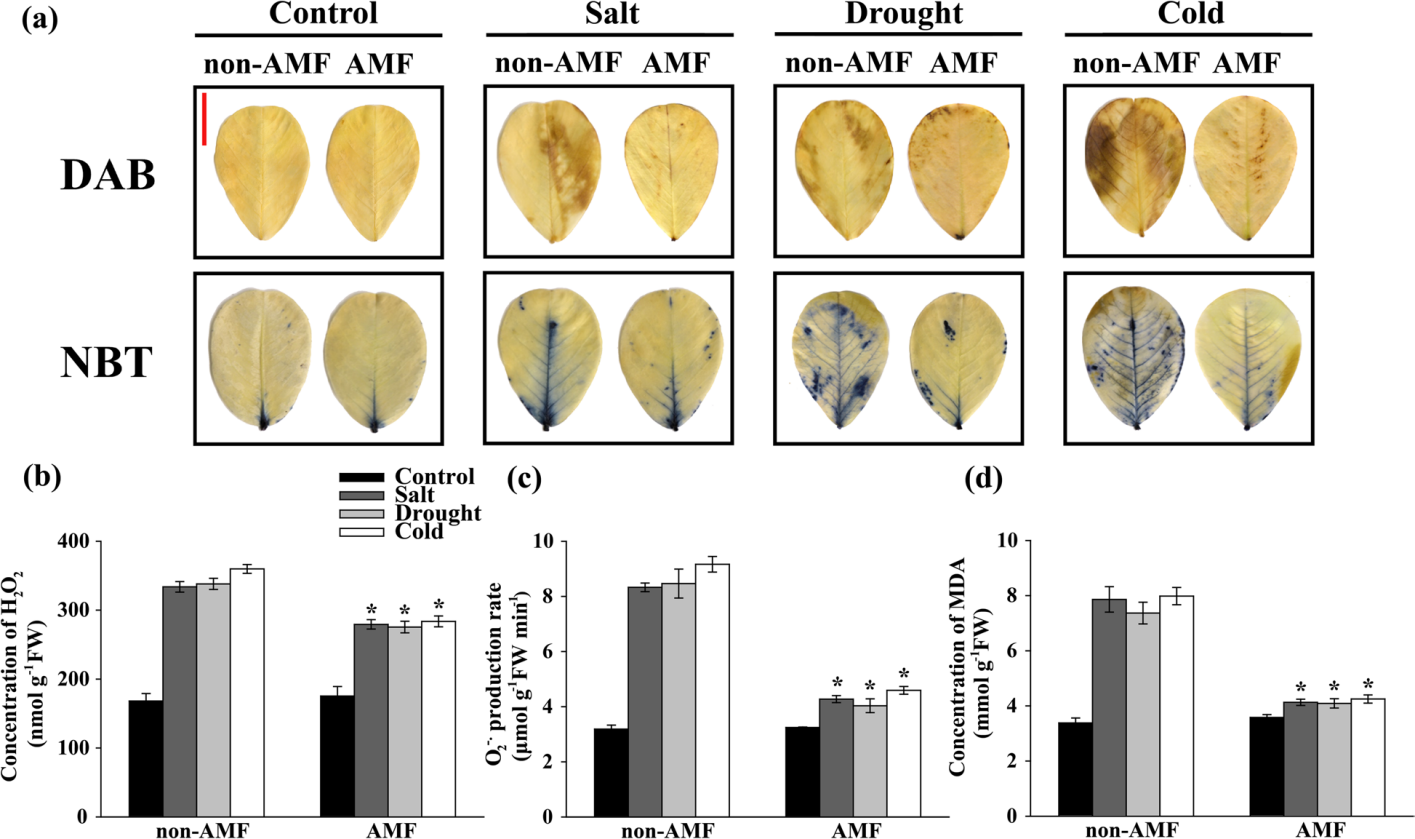
Effect of AMF on the accumulations of H_2_O_2_, O_2_^-.^, and concentration of MDA in the third fully expanded leaves of peanut plants under stressful conditions. The peanut seeds were inoculated with/without AMF before exposing to normal/salt/drought/cold growth conditions. On the 8^th^ day after the onset of stress treatments, the leaves were excised, and the histochemical staining of (**a**) H_2_O_2_ (DAB staining) and O_2_^-.^ (NBT staining) were performed. Vertical bar = 1 cm. Meanwhile, the leaves were collected and the (**b**) concentration of H_2_O_2_, (**c**) O_2_^-.^ production rate, and (**d**) concentration of MDA were measured. Bars represent the mean values of three biological replicates with standard deviation; asterisks indicate a significant difference in comparison to non-AMF according to Tukey's test (*P* < 0.05)

We then detected the accumulation of ROS in peanut leaves. The histochemical localization of H_2_O_2_ was conducted by DAB staining. Salt, drought, and cold stress induced H_2_O_2_ accumulation in peanut leaves whereas the H_2_O_2_ concentration was much lower in AMF-treated plants under stressful conditions. The accumulation of O_2_^-.^ was detected using NBT staining method where salt, drought, and cold induced higher level of O_2_^-.^; however, AMF-treated plants largely reduced the accumulation of O_2_^-.^ under stressful conditions (Fig. 5a). In keeping with these results, the quantitative data further showed that AMF significantly reduced the concentration of H_2_O_2_ by 16.31, 18.54, and 21.16%, respectively, under salt, drought, and cold stress (Fig. 5b). Additionally, a significant reduction of O_2_^-.^ production rate was observed by 48.69, 52.33, and 49.87%, respectively, in AMF-treated leaves under salt, drought, and cold conditions (Fig. 5c). In line with the ROS data, AMF-treated plants significantly reduced the concentration of MDA by 47.46, 44.45, and 46.75%, respectively, in peanut leaves under salt, drought, and cold stress (Fig. 5d).

**Fig. 6:**
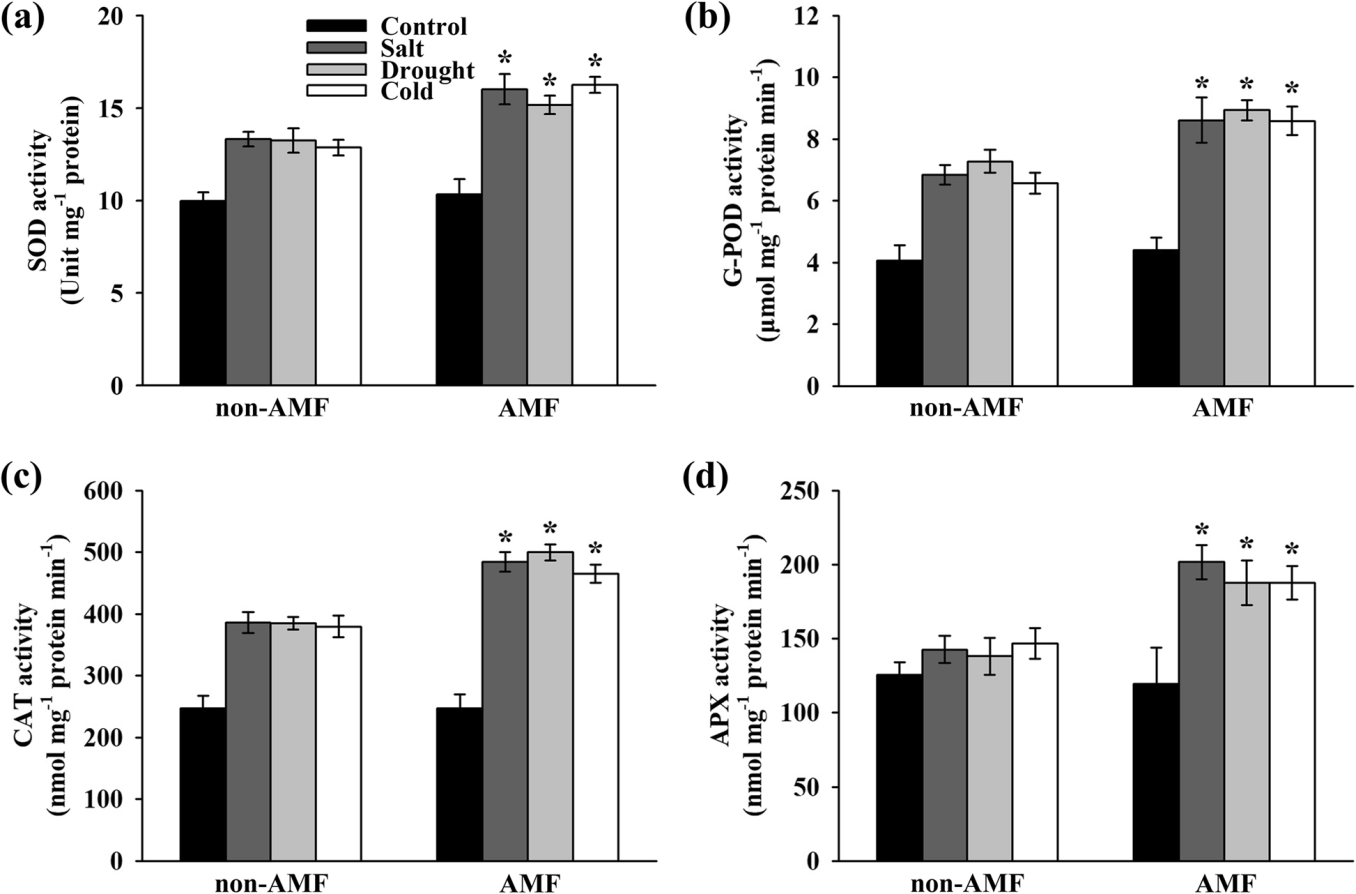
Effect of AMF on antioxidant enzymes in the third fully expanded leaves of peanut plants under stressful conditions. The peanut seeds were inoculated with/without AMF before exposing to normal/salt/drought/cold growth conditions. The activities of (**a**) superoxide dismutase (SOD), (**b**) guaiacol peroxidase (G-POD), (**c**) catalase (CAT), and (**d**) ascorbate peroxidase (APX) were measured on the 8^th^ day after the onset of stress treatments. Bars represent the mean values of three biological replicates with standard deviation; asterisks indicate a significant difference in comparison to non-AMF according to Tukey's test (*P* < 0.05)

Inoculation with AMF did not show any significant differences in activities of antioxidant enzymes including SOD, G-POD, CAT, and APX in peanut leaves under normal growth conditions. In contrast, salt, drought, and cold stress induced the activities of these antioxidant enzymes in varying degrees (Fig. 6). Importantly, AMF-treated plants resulted in a significant increase in SOD activity by 20.18, 14.51, and 26.27% (Fig. 6a), G-POD activity by 25.82, 22.81, and 30.51% (Fig. 6b), CAT activity by 25.52, 29.76, and 22.51% (Fig. 6c), and APX activity by 41.24, 35.89, and 27.89% (Fig. 6d), respectively, under salt, drought, and cold stress compared to their non-treated control.

### Effects of AMF on metabolic profile of peanut roots under environmental stress

**Fig. 7:**
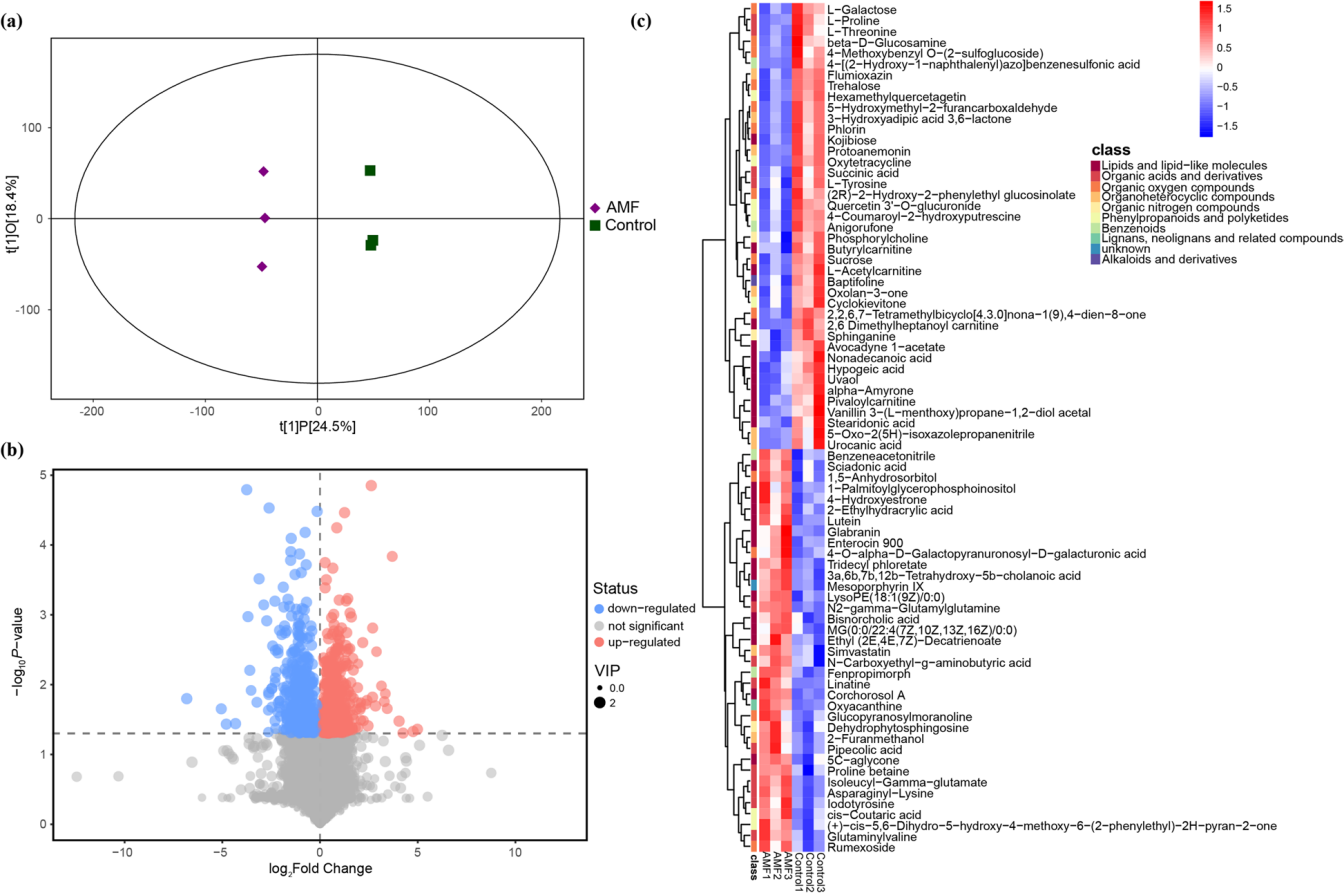
(**a**) OPLS-DA models, (**b**) volcano plots, and (**c**) heat map showing the differential metabolites with MS2 based on the non-target metabolomics in the peanut root samples of “AMF vs Control”

Metabolites differed significantly between treatments from the AMF inoculation and those from the non-inoculated control under both normal-growth and environmental stress conditions. In total, 853, 1163, 375, and 1438 metabolite ion features differed in “AMF vs Control”, “AMF + NaCl vs NaCl”, “AMF + Drought vs Drought”, and “AMF + Cold vs Cold”, respectively (Table S1). OPLS-DA models of the metabolites exhibited a great goodness-of-fit (*R*^*2*^*X*) and high predictability (*Q*^*2*^), with 0.429 and 0.769 in “AMF vs Control” (Fig. 7a), 0.448 and 0.816 in “AMF + NaCl vs NaCl” (Fig. S4a), 0.443 and 0.451 in “AMF + Drought vs Drought” (Fig. S5a), and 0.516 and 0.78 in “AMF + Cold vs Cold” (Fig. S6a). The volcano plot suggested that there were similar up-regulated and down-regulated metabolites in “AMF vs Control” (Fig. 7b) and “AMF + Drought vs Drought” (Fig. S5b); however, more compounds increased in “AMF + NaCl vs NaCl” (Fig. S4b) whereas more compounds decreased in “AMF + Cold vs Cold” (Fig. S6b). Furthermore, heat maps were constructed to obtain an obvious profile of metabolites between the treatments. A total of 78, 113, 35, and 130 metabolites with MS2 identifications, differed significantly in “AMF vs Control” (Fig. 7c), “AMF + NaCl vs NaCl” (Fig. S4c), “AMF + Drought vs Drought” (Fig. S5c), and “AMF + Cold vs Cold” (Fig. S6c), respectively (VIP >1.0 and *P*<0.05). Strikingly, these differential metabolites could be mainly sorted as organic acids.

**Fig. 8:**
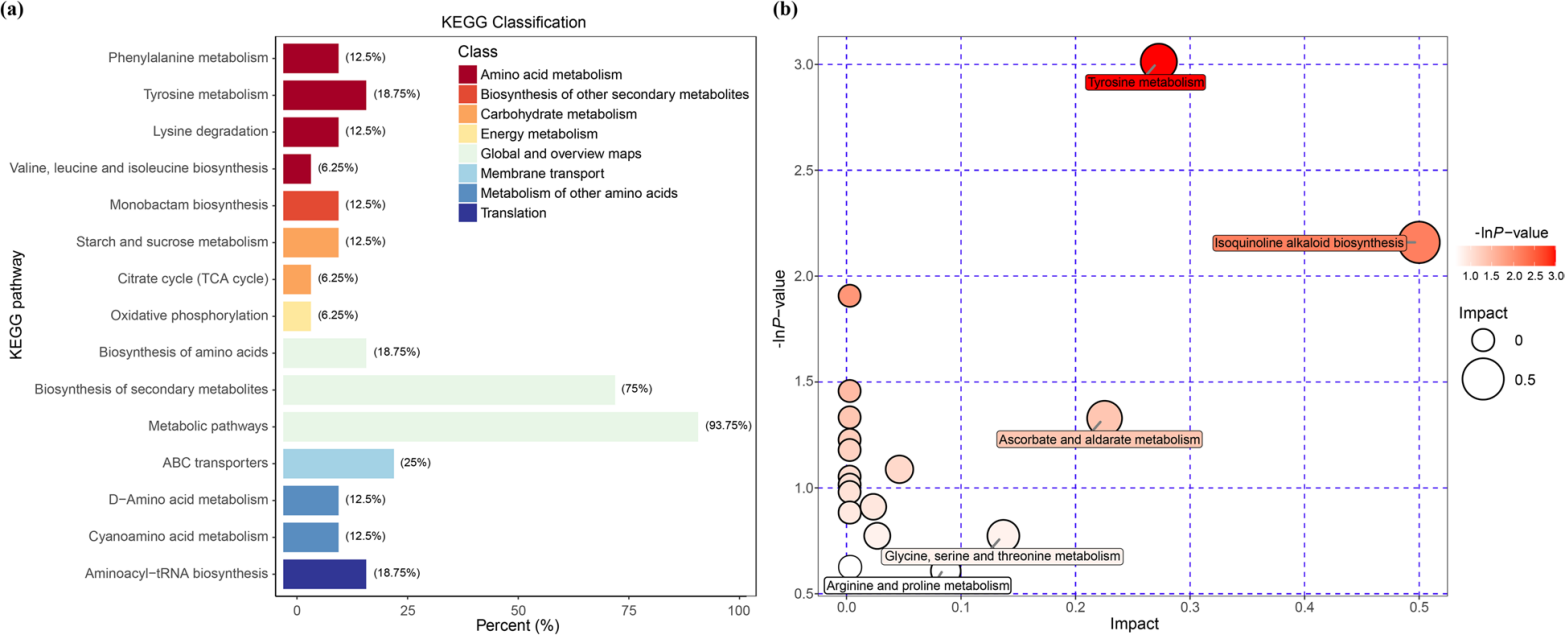
(**a**) KEGG classification and (**b**) biosynthetic pathway analysis based on the non-target metabolomics in the peanut root samples of “AMF vs Control”

To gain further insights into the influence of AMF on secondary metabolite pathway, the differentially abundant metabolites (based on KEGG database) have been determined. In “AMF vs Control”, pathways associated with amino acid metabolism showed the greatest degree of alteration (Fig. 8a). In “AMF + NaCl vs NaCl”, the numbers of pathways regarding amino acid and carbohydrate metabolism were remarkably high (Fig. S7). Similarly, In “AMF + Drought vs Drought” (Fig. S8) and “AMF + Cold vs Cold” (Fig. S9), pathways associated with amino acid and lipid metabolism possessed higher degree of alteration. The biosynthetic pathway analysis further demonstrated these observations where tyrosine metabolism and isoquinoline alkaloid biosynthesis showed significant differences in “AMF vs Control” (Fig. 8b).

### Effects of AMF on osmolytes accumulation under environmental stress

We then determined the role of AMF on osmolytes accumulation in peanut plants. Under normal-growth conditions, no significant difference was observed between AMF-inoculated and non-inoculated leaves. AMF only significantly increased the concentration of total soluble sugar in stems while significantly reduced the concentrations of sucrose and free amino acids in roots compared with control (Table 1). Under salt stress, AMF significantly decreased the concentration of total soluble sugar by 17.52% in leaves while significantly increased the concentration of total soluble sugar by 40.75 and 75.21% in stems and roots, respectively. Moreover, AMF significantly increased the concentration of sucrose by 35.20, 42.37, and 38.18% in leaves, stems, and roots, respectively. Strikingly, the changes of the concentration of free amino acids differed in leaves, stems, and roots when comparing AMF-treated plants to non-treated control. In addition, AMF dramatically increased the concentrations of total soluble sugar, sucrose, and free amino acids by 14.70, 30.29, and 15.86%, respectively in leaves and 19.43, 34.24, and 7.08%, respectively in stems under drought stress, while with little changes in roots. Notably, AMF significantly reduced the accumulation of total soluble sugar by 14.41 and 12.27%, sucrose by 27.78 and 21.14%, and free amino acids by 11.68 and 8.73%, respectively in leaves and stems under cold stress. Conversely, the concentrations of these osmolytes were substantially induced in roots (Table 1).

**Table 1:** Effect of AMF on concentrations of total soluble sugar, sucrose, and free amino acids in leaves, stems, and roots of peanut plants under environmental stress

**Treatment**	**Position**	**Total soluble sugar (mg g**^**−1**^** DW)**	**Sucrose** **(mg g**^**−1**^** DW)**	**Free amino acids (mg 100 g**^**−1**^** DW)**
Control	Leaf	56.90 ± 3.31e	23.11 ± 0.19d	85.00 ± 2.80d
AMF		58.84 ± 3.38de	25.88 ± 0.37cd	84.87 ± 2.94d
NaCl		100.45 ± 5.76b	24.26 ± 2.48cd	110.55 ± 5.77c
AMF + NaCl		82.85 ± 2.98c	32.80 ± 4.63b	118.69 ± 4.47c
Drought		56.52 ± 1.31e	22.83 ± 1.28d	75.15 ± 3.22e
AMF + Drought		64.83 ± 0.51d	29.75 ± 3.29bc	87.06 ± 1.51d
Cold		123.70 ± 2.95a	47.37 ± 4.69a	151.18 ± 4.96a
AMF + Cold		105.88 ± 3.31b	34.21 ± 1.61b	133.51 ± 3.09b
Control	Stem	65.34 ± 5.81d	21.94 ± 1.45f	46.66 ± 6.19f
AMF		96.43 ± 2.93c	23.50 ± 2.19ef	46.59 ± 2.35f
NaCl		48.61 ± 3.78e	24.31 ± 1.80ef	81.92 ± 5.64c
AMF + NaCl		68.42 ± 1.70d	34.61 ± 2.39cd	69.63 ± 4.70d
Drought		104.62 ± 6.01c	28.87 ± 2.27de	59.44 ± 1.58e
AMF + Drought		124.95 ± 4.55a	38.75 ± 2.40c	63.65 ± 1.13de
Cold		129.27 ± 4.70a	61.08 ± 4.58a	130.48 ± 3.07a
AMF + Cold		113.40 ± 3.77b	48.17 ± 2.96b	119.09 ± 1.92b
Control	Root	55.78 ± 1.19e	26.25 ± 1.49d	73.08 ± 1.43e
AMF		55.84 ± 2.82e	20.24 ± 2.48e	66.11 ± 3.54f
NaCl		71.77 ± 3.82d	29.70 ± 0.59d	93.68 ± 2.68c
AMF + NaCl		125.75 ± 4.91a	41.04 ± 0.82c	108.66 ± 3.92b
Drought		89.48 ± 6.32c	46.02 ± 1.59bc	71.75 ± 2.82e
AMF + Drought		86.12 ± 2.21c	41.35 ± 0.48c	64.47 ± 1.48f
Cold		115.25 ± 2.28b	48.19 ± 1.04ab	85.44 ± 0.55d
AMF + Cold		129.72 ± 6.75a	51.64 ± 5.02a	157.84 ± 1.44a

Data are presented as the means ± standard deviation (SD) of three replications. Different letters in the same column of the same position indicate significant differences among treatments (*P* < 0.05)

We further evaluated the accumulation and distribution of K^+^ and Na^+^ in peanut roots and leaves. Salt, drought, and cold stress resulted in a decline in K^+^ content in peanut roots; however, inoculation with AMF significantly increased the K^+^ content by 28.2, 24.81, and 34.38%, respectively (Fig. S3a). In contrast, salt, drought, and cold stress dramatically increased the Na^+^ content, especially under salt stress in peanut roots. Again, treatment with AMF significantly reduced the Na^+^ content by 23.44, 30.76, and 26.69%, respectively (Fig. S3b). Interestingly, AMF significantly increased the K^+^: Na^+^ ratio by 67.17, 80.99, and 83.55%, respectively, under salt, drought, and cold stress (Fig. S3c). Similarly, the K^+^ content in AMF-treated peanut leaves was significantly increased by 15.11, 14.15, and 18.44%, respectively, under salt, drought, and cold stress (Fig. S3d). Meanwhile, even though environmental stress induced the Na^+^ content to a large extent, inoculation with AMF showed a significant decrease in Na^+^ content by 44.62, 42.62, and 36.96%, respectively, in peanut leaves under salt, drought, and cold conditions (Fig. S3e). Ultimately, AMF significantly increased the K^+^: Na^+^ ratio by 107.88, 100.19, and 89.68%, respectively, in peanut leaves under salt, drought, and cold stress (Fig. S3f).

## Discussion

AMF play essential roles in plant resistance subjected to various environmental stress and could potentially help farmers reduce the crop yield losses in the changing global climate [32, 35, 71, 72]. Here we investigate, to the best of our knowledge for the first time, the multifaceted roles of a new combination of AMF species in the alleviation of salt, drought, and cold stress in peanut plants. We therefore emphasize the importance of integrating AMF in legume crop production for designing proper strategies against environmental stress under the changing natural ecosystems.

AMF-treated peanut showed obviously improved adaptability to salt, drought, and cold stress, respectively, which was largely dependent on the improved plant growth in both above-ground (Fig. 1) and below-ground (Fig. S2) parts of the plants. The outcomes of AMF symbiosis are suggested to be nutrient acquisition, hence, the soil nutrient availability and solubility are strongly influenced by AMF inoculation [32, 73, 74]. As a result, the enhanced peanut plant growth become the capital of plants in stress adaptation [31, 34]. We previously observed that AMF-treated peanut plants exhibited higher accumulation of nitrogen, phosphorus, and potassium [19]. In line with our previous findings, we further showed that AMF significantly enhanced the root volume, total root length, and root surface area, but not root average diameter under multiple environmental stress (Fig. S2). Therefore, the strengthened plant growth observed in the present study might be partially related to the improved plant nutrition absorption through the enhanced radicular absorption area.

Apart from root architecture, extensive attention has been paid to the photosystem. The increased photosynthetic carbon fixation efficiency could contribute to the ultimate biomass production of a plant [75–77]. We observed that the AMF-enhanced shoot dry weight (Fig. 1c) was associated with the increased Pn (Fig. 2a) in plants grown under stressful conditions, which was in good agreement with the previous studies. Notably, the changes of Gs were consistent with Pn (Fig. 2b), hinting a possibility that the depression of Pn might be attributed to stomatal limitations under environmental stress [78, 79]. Thus, AMF could enhance the photosynthesis through promoting stomatal opening. It is therefore plausible that AMF alleviated salt, drought, and cold-induced peanut growth reduction through enhancing the photosynthesis in the leaves.

Leaf chlorophyll fluorescence imaging has long been recognized as a non-invasive and non-destructive method to monitor the potential injury of photosynthetic apparatus [80–82]. In this study, we have provided evidence that AMF increased the stress-induced reduction of Fv/Fm (Fig. 3a & c), suggesting that AMF could reduce the damage to the photosystem under stressful conditions [83, 84]. Consistent with the chlorophyll fluorescence data, AMF also induced the leaf chlorophyll content under environmental stress, further demonstrating the indispensable role of AMF in protecting the peanut leaf photosynthetic pigments (Fig. 3b). AMF also induced the qP under stressful conditions (Fig. 3d), suggesting that AMF could improve the utilization ratio of light energy through enhancing the electron transport activity. ΦPSII corresponds to the fraction of energy that is photochemically converted in PSII [85], therefore, it seems that AMF led to an increased ability of peanut leaf to use light energy for photochemistry (Fig. 3e). Thus, we deduce that AMF play a dominant role in reducing the injury of peanut photosynthetic apparatus under environmental constraints.

The data presented here also suggest that AMF contribute to the maintaining of intact ultrastructure of chloroplast thylakoids and mitochondria (Fig. 4). Literatures indicated that the reaction centers of PSI and PSII in thylakoids are the major source of ROS when plants were exposed to stressful conditions [50, 86, 87]. Consistent with these findings, the scavenging effect of AMF on ROS in peanut leaves was also observed (Figs. 5 and 6). Therefore, these results, together with the previous reports, demonstrate that AMF play an essential role in protecting peanut photosystem, possibly through regulating the photosynthetic electron transport chain. To achieve greater insight into the role of AMF in the control of the adverse effects of ROS, we determined the accumulation of H_2_O_2_ and O_2_^-.^ and clearly demonstrated that inoculation with AMF inhibited the accumulation of ROS in peanut leaves under environmental stress (Fig. 5 b & c). Interestingly, treatment with AMF showed not significant increases in the concentrations of H_2_O_2_ and O_2_^-.^ under normal growth conditions. The histochemical staining data further corroborated these observations (Fig. 5a), pointing out the profound role of AMF in elimination of ROS to reduce toxic radicals in peanut leaves. It is reported that AMF inoculated plants also exhibited enhanced activities of antioxidant enzymes under stressful conditions [30, 41, 88]. In line with the previous findings, AMF-treated plants exhibited dramatic increases in the activities of antioxidant enzymes including SOD, G-POD, CAT, and APX (Fig. 6). Moreover, the concentration of MDA was dramatically decreased by AMF (Fig. 5d), thereby providing greater stability to plasma membrane.

Osmotic adjustment is one of the plant strategies to minimize the adverse effects of environmental stress [89, 90]. Apart from osmoregulation, the metabolisms of organic acids may also act as ROS scavengers and signalling molecules in overcoming abiotic stress [91, 92]. In agreement with these findings, the reduced accumulation of ROS and MDA (Fig. 5) might be the partially due to the induction of organic acids in AMF-inoculated peanut plants under stressful conditions, as evidenced by the metabolomics data. The early effects of salt and drought stress are similar because they both impose osmotic stress [93, 94]. Additionally, the symbiosis of AMF could in principle ameliorate the hydraulic properties of their host plants [29, 35]. Consistent with the earlier reports [95–97], we observed that AMF dramatically enhanced the concentrations of total soluble sugar, sucrose, and free amino acids under salt and drought stress with only a few exceptions (Table 1). It is well accepted that the deficiency of nutrient elements strongly affects the type and amount of secondary metabolites [98, 99], with a subsequent negative impact on plant abiotic stress resistance [90, 100]. Particularly, amino acids have been taken as vital precursors in a serious of secondary metabolites [101, 102]. Our metabolomics data further confirmed the results of the quantitative analysis where pathways associated with amino acids showed the greatest degree of alteration in all comparisons. Notably, under normal growth conditions, amino acids such as proline and threonine were highly accumulated by AMF inoculation (Fig. 7) and therefore provide theoretical basis for alleviating the abiotic stress to come. On the basis of the obtained information, we deduce that the metabolism of amino acids and organic acids might be the vital hub in AMF-induced peanut resistance to salt, drought, and cold stress. Taken together, the results of the metabolomics analysis have provided new insights into the profound role of AMF in osmoregulation of their host plants to alleviate abiotic stress.

Plants exposed to environmental stress especially salt stress always suffer from Na^+^ toxicity and K^+^ deficiency [103–105]. Plants depend to a great extent on the homeostasis of K^+^: Na^+^ ratio, since the higher ratio of K^+^: Na^+^ could help to efflux of Na^+^ from cells as well as induction of osmotic adjustment [29, 106, 107]. In this study, AMF significantly reduced the excessive accumulation of Na^+^ while induced the content of K^+^ in both roots and leaves of peanut plants under salt, drought, and cold stress (Fig. S3). Hence, the maintenance of K^+^: Na^+^ homeostasis by AMF might facilitate the uptake of K^+^ while prevent the absorption of Na^+^ in peanut roots, and consequently contributes to alleviating abiotic stress.

## Conclusions

**Fig. 9:**
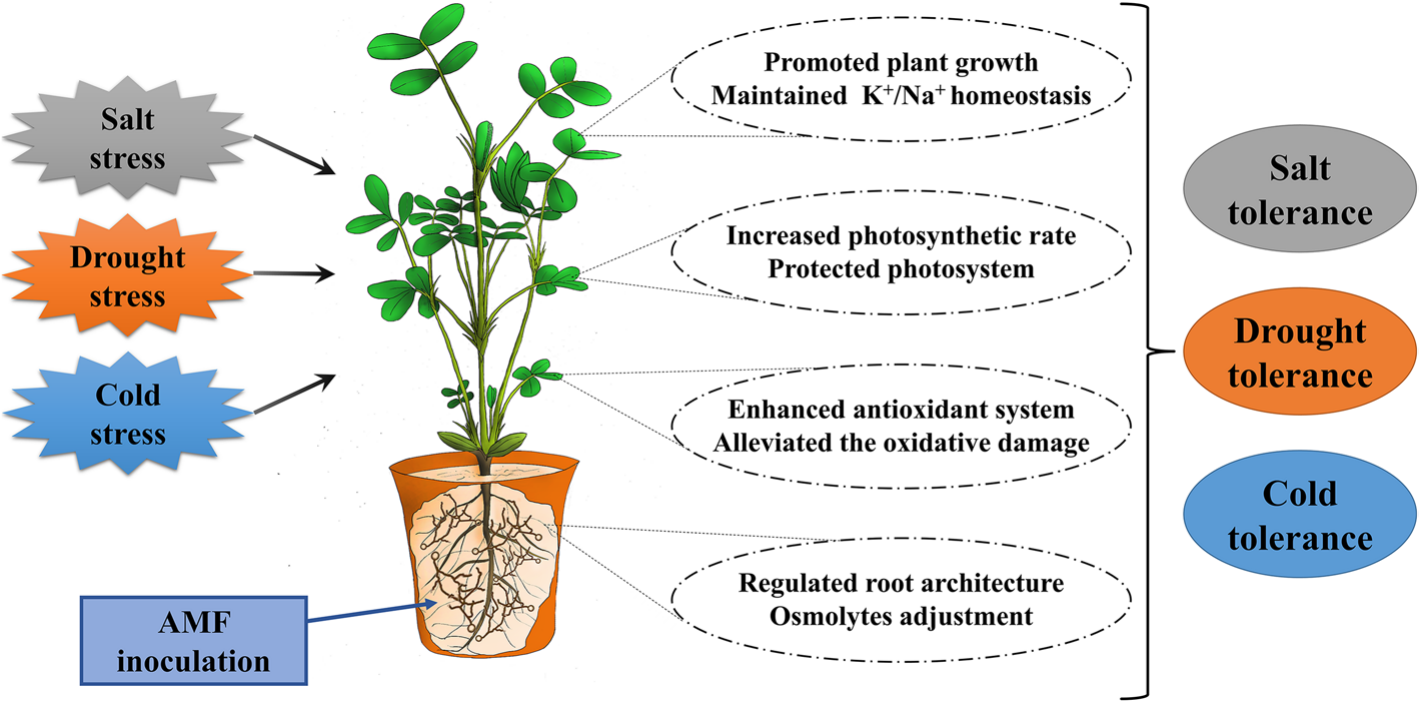
The working model illustrating the mechanisms of AMF in alleviating peanut salt, drought, and cold stress

The present study showed promising effects of AMF on peanut environmental stress responses. Inoculation with AMF species *Rhizophagus irregularis* SA, *Rhizophagus clarus* BEG142, *Glomus lamellosum* ON393, and *Funneliformis mosseae* BEG95 (1: 1: 1: 1, w/w/w/w) improved environmental stress tolerance in terms of plant growth, leaf water status, and root architecture. In addition, AMF increased the Pn by protecting the integrity of photosystem and alleviated the oxidative damage by enhancing the antioxidant system under stressful conditions. Notably, the regulation of osmolytes accumulation including organic acids and amino acids as well as the maintenance of K^+^: Na^+^ homeostasis in both roots and leaves of peanut also play novel roles in alleviating salt, drought, and cold stress (Fig. 9). The present work promotes a more comprehensive understanding of the physiological and metabolomic mechanisms of AMF in combating peanut environmental stress. Further studies pertaining to the molecular responses of AMF under climate change scenarios are warranted to fully elucidate the interspecific interactions between AMF and legume crop species.

## Supplementary Information

**Additional file 1: Fig. S1.** (a) Schematic representation of experimental design and treatments. At 40 days after emergence, both AMF-inoculated and non-inoculated plants were exposed to salt, drought, or cold stress for 7 successive days before measurements and samples were taken. (b) The mycorrhization status of the AMF-inoculated peanut roots (200×). **Fig. S2. **Effect of AMF on root morphology of peanut plants under stressful conditions. The peanut seeds were inoculated with/without AMF before exposing to normal/salt/drought/cold growth conditions. The roots were excised, washed thoroughly, and scanned on the 8^th^ day after the onset of stress treatments. (a) One representative picture is shown for each treatment. The (b) root volume, (c) total root length, (d) root average diameter, and (e) root surface area were determined. Bars represent the mean values of three biological replicates with standard deviation; asterisks indicate a significant difference in comparison to non-AMF according to Tukey's test (*P* < 0.05). **Fig. S3. **Effect of AMF on the accumulations of K^+^, Na^+^ and the K^+^: Na^+^ ratio of roots (a-c) and leaves (d-f) in peanut plants under stressful conditions. The peanut seeds were inoculated with/without AMF before exposing to normal/salt/drought/cold growth conditions. The root and leaf samples were taken on the 8^th^ day after the onset of stress treatments. Bars represent the mean values of three biological replicates with standard deviation; asterisks indicate a significant difference in comparison to non-AMF according to Tukey's test (*P* < 0.05). **Fig. S4 .**OPLS-DA models (a), volcano plots (b), and heat map showing the differential metabolites with MS2 (c) based on the non-target metabolomics in the peanut root samples of “AMF + NaCl vs NaCl”. **Fig. S5. **OPLS-DA models (a), volcano plots (b), and heat map showing the differential metabolites with MS2 (c) based on the non-target metabolomics in the peanut root samples of “AMF + Drought vs Drought”. **Fig. S6. **OPLS-DA models (a), volcano plots (b), and heat map showing the differential metabolites with MS2 (c) based on the non-target metabolomics in the peanut root samples of “AMF + Cold vs Cold”. **Fig. S7. **KEGG classification (a) and biosynthetic pathway analysis (b) based on the non-target metabolomics in the peanut root samples of “AMF + NaCl vs NaCl”. **Fig. S8. **KEGG classification based on the non-target metabolomics in the peanut root samples of “AMF + Drought vs Drought”. **Fig. S9. **KEGG classification (a) and biosynthetic pathway analysis (b) based on the non-target metabolomics in the peanut root samples of “AMF + Cold vs Cold”.

**Additional file 2:** **Table S1.** Differentially expressed metabolites.

## Data Availability

All data generated or analyzed during this study are included in this published article (and its supplementary information files).

## Abbreviations

AMF: Arbuscular Mycorrhizal Fungi
ANOVA: Analysis of variance
APX: Ascorbate peroxidase
CAT: Catalase
Ci: Intercellular CO_2_ concentration
DAB, 3: 3-Diaminobenzidine
DW: Dry weight
Fm: Maximal fluorescence
Fm’: Light-adapted maximum fluorescence
Fo: Minimal fluorescence emission signal
Fv/Fm: Maximal photochemical efficiency of photosystem II
FW: Fresh weight
Fs: Steady-state fluorescence yield
G-POD: Guaiacol peroxidase
Gs: Stomatal conductance
H_2_O_2_: Hydrogen peroxide
K: Potassium
KEGG: Kyoto Encyclopedia of Genes and Genomes
MDA: Malondialdehyde
Na: Sodium
NBT: Nitro blue tetrazolium
O_2_^−.^: Superoxide anion
OPLS-DA: Orthogonal projections to latent structures-discriminate analysis
PAM: Pulse amplitude modulated
Pn: The net photosynthetic rate
PPFD: Photosynthetic photon flux density
qP: Photochemical quenching coefficient
REC: Relative electrolyte conductivity
ROS: Reactive oxygen species
RWC: Relative water content
SOD: Superoxide dismutase
TBA: 2-thiobarbituric acid
Tr: Transpiration rate
TW: Turgid weight
ΦPSII: The quantum yield of PSII photochemistry

## References

[1] Raja V, Majeed U, Kang H, Andrabi KI, John R (2017). Abiotic stress: Interplay between ROS, hormones and MAPKs. Environ Exp Bot.

[2] Farooq MA, Niazi A, Akhtar J, Ullah S, Farooq M, Souri Z, Karimi N, Rengel Z (2019). Acquiring vontrol: The evolution of ROS-induced oxidative stress and redox signaling pathways in plant stress responses. Plant Physiol Biochem.

[3] Godoy F, Olivos-Hernández K, Stange C, Handford M (2021). Abiotic stress in crop species: improving tolerance by applying plant metabolites. Plants.

[4] Gilroy S, Suzuki N, Miller G, Choi W-G, Toyota M, Devireddy AR (2014). A tidal wave of signals: calcium and ROS at the forefront of rapid systemic signaling. Trends Plant Sci.

[5] Zhao C, Wang P, Si T, Hsu C-C, Wang L, Zayed O, Yu Z, Zhu Y, Dong J, Tao WA (2017). MAP Kinase cascades regulate the cold response by modulating ICE1 protein stability. Dev Cell.

[6] Baxter A, Mittler R, Suzuki N (2014). ROS as key players in plant stress signalling. J Exp Bot.

[7] Xia X, Zhou Y, Shi K, Zhou J, Foyer CH, Yu J (2015). Interplay between reactive oxygen species and hormones in the control of plant development and stress tolerance. J Exp Bot.

[8] Choudhury FK, Rivero RM, Blumwald E, Mittler R (2017). Reactive oxygen species, abiotic stress and stress combination. Plant J.

[9] Miller G, Schlauch K, Tam R, Cortes D, Torres MA, Shulaev V, Dangl JL, Mittler R (2009). The Plant NADPH Oxidase RBOHD Mediates Rapid Systemic Signaling in Response to Diverse Stimuli. Sci Signal.

[10] Dietz K-J, Mittler R, Noctor G (2016). Recent progress in understanding the role of reactive oxygen species in plant cell signaling. Plant Physiol.

[11] Xie W, Zhang K, Wang X, Zou X, Zhang X, Yu X, Wang Y, Si T (2022). Peanut and cotton intercropping increases productivity and economic returns through regulating plant nutrient accumulation and soil microbial communities. BMC Plant Biol.

[12] Dowling A, Sadras O, Roberts V, Doolette P, Zhou A, Denton Y (2021). Legume-oilseed intercropping in mechanised broadacre agriculture-a review. Field Crops Res.

[13] Ferguson Brett J, Mens C, Hastwell April H, Zhang M, Su H, Jones Candice H, Chu X, Gresshoff Peter M (2019). Legume nodulation: The host controls the party. Plant Cell Environ.

[14] IPCC. Climate Change 2007: The Physical Science Basis. In: Olomon S, Qin D, Manning M, et al., editors. Contribution of Working Group I to the Fourth Assessment Report of the Intergovernmental Panel on Climate Change. Cambridge & New York: Cambridge University Press; 2007.

[15] IPCC. Climate Change 2014: Mitigation of Climate Change. In: Edenhofer OR, Pichs-Madruga Y, Sokona E, Farahani S, Kadner K, Seyboth A, et al., editors. Contribution of Working Group III to the Fifth Assessment Report of the Intergovernmental Panel on Climate Change. Cambridge & New York: Cambridge University Press; 2014.

[16] Considine MJ, Siddique KHM, Foyer CH (2017). Nature’s pulse power: legumes, food security and climate change. J Exp Bot.

[17] Cao J, An Q, Zhang X, Xu S, Si T, Niyogi D (2021). Is satellite Sun-Induced Chlorophyll Fluorescence more indicative than vegetation indices under drought condition?. Sci Total Environ.

[18] Détain A, Bhowmik P, Leborgne-Castel N, Ochatt S (2022). Latest biotechnology tools and targets for improving abiotic stress tolerance in protein legumes. Environ Exp Bot.

[19] Qin W, Yan H, Zou B, Guo R, Ci D, Tang Z, Zou X, Zhang X, Yu X, Wang Y (2021). Arbuscular mycorrhizal fungi alleviate salinity stress in peanut: Evidence from pot-grown and field experiments. Food Energy Secur.

[20] Aninbon C, Jogloy S, Vorasoot N, Nuchadomrong S, Senawong T, Holbrook CC, Patanothai A (2016). Effect of mid season drought on phenolic compounds in peanut genotypes with different levels of resistance to drought. Field Crops Res.

[21] Si T, Wang X, Zhou Y, Zhang K, Xie W, Yuan H, Wang Y, Sun Y (2022). Seed yield and quality responses of oilseed crops to simulated nitrogen deposition: A meta-analysis of field studies. GCB Bioenergy.

[22] El-Akhal MR, Rincón A, Coba de la Peña T, Lucas MM, El Mourabit N, Barrijal S, Pueyo JJ (2012). Effects of salt stress and rhizobial inoculation on growth and nitrogen fixation of three peanut cultivars. Plant Biol.

[23] Palmero F, Carcedo AJP, Haro RJ, Bigatton ED, Salvagiotti F, Ciampitti IA (2022). Modeling drought stress impacts under current and future climate for peanut in the semiarid pampas region of Argentina. Field Crops Res.

[24] Zhang G, Dai L, Ding H, Ci D, Ning T, Yang J, Zhao X, Yu H, Zhang Z (2020). Response and adaptation to the accumulation and distribution of photosynthetic product in peanut under salt stress. J Integr Agric.

[25] Sun R, Zheng H, Yin S, Zhang X, You X, Wu H, Suo F, Han K, Cheng Y, Zhang C (2022). Comparative study of pyrochar and hydrochar on peanut seedling growth in a coastal salt-affected soil of Yellow River Delta China. Sci Total Environ.

[26] Zhang H, Jiang C, Lei J, Dong J, Ren J, Shi X, Zhong C, Wang X, Zhao X, Yu H (2022). Comparative physiological and transcriptomic analyses reveal key regulatory networks and potential hub genes controlling peanut chilling tolerance. Genomics.

[27] Hussain N, Sarwar G, Schmeisky H, Al-Rawahy S, Ahmad M. Salinity and drought management in legume crops. In: Climate change and management of cool season grain legume crops. Dordrecht: Springer; 2010. p. 171–91. 10.1007/978-90-481-3709-1_10.

[28] Ye L, Zhao X, Bao E, Cao K, Zou Z (2019). Effects of Arbuscular Mycorrhizal Fungi on watermelon growth, elemental uptake, antioxidant, and photosystem II activities and stress-response gene expressions under salinity-alkalinity stresses. Front Plant Sci.

[29] Aroca R, Porcel R, Ruiz-Lozano JM (2007). How does arbuscular mycorrhizal symbiosis regulate root hydraulic properties and plasma membrane aquaporins in Phaseolus vulgaris under drought, cold or salinity stresses?. New Phytol.

[30] Begum N, Ahanger MA, Zhang L (2020). AMF inoculation and phosphorus supplementation alleviates drought induced growth and photosynthetic decline in Nicotiana tabacum by up-regulating antioxidant metabolism and osmolyte accumulation. Environ Exp Bot.

[31] Qiu Q, Bender SF, Mgelwa AS, Hu Y (2022). Arbuscular mycorrhizal fungi mitigate soil nitrogen and phosphorus losses: A meta-analysis. Sci Total Environ.

[32] Zhang S, Lehmann A, Zheng W, You Z, Rillig MC (2019). Arbuscular mycorrhizal fungi increase grain yields: a meta-analysis. New Phytol.

[33] Larimer AL, Clay K, Bever JD (2014). Synergism and context dependency of interactions between arbuscular mycorrhizal fungi and rhizobia with a prairie legume. Ecology.

[34] Cabral C, Ravnskov S, Tringovska I, Wollenweber B (2016). Arbuscular mycorrhizal fungi modify nutrient allocation and composition in wheat (*Triticum aestivum* L.) subjected to heat-stress. Plant Soil.

[35] Lenoir I, Fontaine J, Lounès-Hadj Sahraoui A (2016). Arbuscular mycorrhizal fungal responses to abiotic stresses: A review. Phytochemistry.

[36] Ci D, Tang Z, Ding H, Cui L, Zhang G, Li S (2021). The synergy effect of arbuscular mycorrhizal fungi symbiosis and exogenous calcium on bacterial community composition and growth performance of peanut (*Arachis hypogaea* L.) in saline alkali soil. J Microbiol.

[37] Nguyen HP, Miwa H, Obirih-Opareh J, Suzaki T, Yasuda M, Okazaki S (2020). Novel rhizobia exhibit superior nodulation and biological nitrogen fixation even under high nitrate concentrations. FEMS Microbiology Ecology.

[38] Becana M, Matamoros MA, Udvardi M, Dalton DA (2010). Recent insights into antioxidant defenses of legume root nodules. New Phytol.

[39] Eroğlu ÇG, Cabral C, Ravnskov S, Bak Topbjerg H, Wollenweber B (2020). Arbuscular mycorrhiza influences carbon-use efficiency and grain yield of wheat grown under pre- and post-anthesis salinity stress. Plant Biol.

[40] Zhu X, Song F, Liu S, Liu F (2016). Role of Arbuscular Mycorrhiza in alleviating salinity stress in wheat (*Triticum aestivum* L.) grown under ambient and elevated CO_2_. J Agron Crop Sci.

[41] Li J, Meng B, Chai H, Yang X, Song W, Li S (2019). Arbuscular Mycorrhizal Fungi alleviate drought stress in C_3_> (*Leymus chinensis*) and C_4_ (*Hemarthria altissima*) grasses via altering antioxidant enzyme activities and photosynthesis. Front Plant Sci.

[42] Neidhardt H (2021). Arbuscular mycorrhizal fungi alleviate negative effects of arsenic-induced stress on crop plants: A meta-analysis. Plants, People, Planet.

[43] Etesami H, Li Z, Maathuis FJM, Cooke J (2022). The combined use of silicon and arbuscular mycorrhizas to mitigate salinity and drought stress in rice. Environ Exp Bot.

[44] Tian S, Guo R, Zou X, Zhang X, Yu X, Zhan Y (2019). Priming with the green leaf volatile (Z)-3-Hexeny-1-yl Acetate enhances salinity stress tolerance in peanut (*Arachis hypogaea* L.) seedlings. Front Plant Sci.

[45] Guo R, Yan H, Li X, Zou X, Zhang X, Yu X, Ci D, Wang Y, Si T (2020). Green leaf volatile (Z)-3-hexeny-1-yl acetate reduces salt stress in peanut by affecting photosynthesis and cellular redox homeostasis. Physiol Plant.

[46] Phillips JM, Hayman DS (1970). Improved procedures for clearing roots and staining parasitic and vesicular-arbuscular mycorrhizal fungi for rapid assessment of infection. Trans Br Mycol Soc.

[47] Kramer DM, Johnson G, Kiirats O, Edwards GE (2004). New fluorescence parameters for the determination of Q_A_ redox state and excitation energy fluxes. Photosynth Res.

[48] Genty B, Briantais J-M, Baker NR (1989). The relationship between the quantum yield of photosynthetic electron transport and quenching of chlorophyll fluorescence. Biochim Biophys Act Subj.

[49] Lichtenthaler HK, Wellburn AR (1983). Determinations of total carotenoids and chlorophylls a and b of leaf extracts in different solvents. Biochem Soc Trans.

[50] Xu F, Zhang D, Zhu F, Tang H, Lv X, Cheng J (2012). A novel role for cyanide in the control of cucumber (*Cucumis sativus* L.) seedlings response to environmental stress. Plant Cell Environ.

[51] Si T, Wang X, Wu L, Zhao C, Zhang L, Huang M, Cai J, Zhou Q, Dai T, Zhu J-K (2017). Nitric oxide and hydrogen peroxide mediate wounding-induced freezing tolerance through modifications in photosystem and antioxidant system in wheat. Front Plant Sci.

[52] Thordal-Christensen H, Zhang Z, Wei Y, Collinge DB (1997). Subcellular localization of H_2_O_2_ in plants. H_2_O_2_ accumulation in papillae and hypersensitive response during the barley-powdery mildew interaction. Plant J.

[53] Willekens H, Chamnongpol S, Davey M, Schraudner M, Langebartels C, Van Montagu M, Inzé D, Van Camp W (1997). Catalase is a sink for H_2_O_2_ and is indispensable for stress defence in C3 plants. EMBO J.

[54] Elstner E, Heupel A (1976). Inhibition of nitrite formation from hydroxylammoniumchloride: a simple assay for superoxide dismutase. Anal Biochem.

[55] Jensen CR, Jacobsen SE, Andersen MN, Núñez N, Andersen SD, Rasmussen L, Mogensen VO (2000). Leaf gas exchange and water relation characteristics of field quinoa (Chenopodium quinoa Willd.) during soil drying. Eur J Agron.

[56] Griffith M, Mclntyre HCH (1993). The interrelationship of growth and frost tolerance in winter rye. Physiol Plant.

[57] Hodges DM, DeLong JM, Forney CF, Prange RK (1999). Improving the thiobarbituric acid-reactive-substances assay for estimating lipid peroxidation in plant tissues containing anthocyanin and other interfering compounds. Planta.

[58] Bradford MM (1976). Rapid and sensitive method for quantitation of microgram quantities of protein utilizing principle of protein-dye binding. Anal Biochem.

[59] Stewart RRC, Bewley JD (1980). Lipid peroxidation associated with accelerated aging of soybean axes. Plant Physiol.

[60] Cakmak I, Marschner H (1992). Magnesium deficiency and high light intensity enhance activities of superoxide dismutase, ascorbate peroxidase, and glutathione reductase in bean leaves. Plant Physiol.

[61] Patra HK, Kar M, Mishra D (1978). Catalase activity in leaves and cotyledons during plant development and senescence. Biochemie Und Physiologie Der Pflanzen.

[62] Nakano Y, Asada K (1981). Hydrogen peroxide is scavenged by ascorbate- specific peroxidase in spinach chloroplasts. Plant Cell Physiol.

[63] Zhang X, Zheng C, Dai T, Cao W, Jiang D (2011). Post-anthesis salt and combination of salt and waterlogging affect distributions of sugars, amino acids, Na^+^ and K^+^ in wheat. J Agron Crop Sci.

[64] Kong X, Luo Z, Dong H, Eneji AE, Li W (2011). Effects of non-uniform root zone salinity on water use, Na^+^ recirculation, and Na^+^ and H^+^ flux in cotton. J Exp Bot.

[65] Buysse JAN, Merckx R (1993). An improved colorimetric method to quantify sugar content of plant tissue. J Exp Bot.

[66] Moore S, Stein WH (1954). A modified ninhydrin reagent for the photometric determination of amino acids and related compounds. J Biol Chem.

[67] De Vos RCH, Moco S, Lommen A, Keurentjes JJB, Bino RJ, Hall RD (2007). Untargeted large-scale plant metabolomics using liquid chromatography coupled to mass spectrometry. Nat Protoc.

[68] Theodoridis G, Gika HG, Wilson ID (2008). LC-MS-based methodology for global metabolite profiling in metabonomics/metabolomics. TrAC Trends in Analytical Chemistry.

[69] Kanehisa M, Goto S (2000). KEGG: Kyoto Encyclopedia of Genes and Genomes. Nucleic Acids Res.

[70] Kanehisa M (2019). Toward understanding the origin and evolution of cellular organisms. Protein Sci.

[71] Branco S, Schauster A, Liao H-L, Ruytinx J (2022). Mechanisms of stress tolerance and their effects on the ecology and evolution of mycorrhizal fungi. New Phytol.

[72] Marro N, Grilli G, Soteras F, Caccia M, Longo S, Cofré N, Borda V, Burni M, Janoušková M, Urcelay C (2022). The effects of arbuscular mycorrhizal fungal species and taxonomic groups on stressed and unstressed plants: a global meta-analysis. New Phytol.

[73] Werner GDA, Kiers ET (2015). Partner selection in the mycorrhizal mutualism. New Phytol.

[74] Thirkell TJ, Charters MD, Elliott AJ, Sait SM, Field KJ (2017). Are mycorrhizal fungi our sustainable saviours? Considerations for achieving food security. J Ecol.

[75] Guo Z, Wang F, Xiang X, Ahammed GJ, Wang M, Onac E, Zhou J, Xia X, Shi K, Yin X (2016). Systemic Induction of Photosynthesis via Illumination of the Shoot Apex Is Mediated Sequentially by Phytochrome B, Auxin and Hydrogen Peroxide in Tomato. Plant Physiol.

[76] Jiang Y, Cheng F, Zhou Y, Xia X, Mao W, Shi K, Chen Z, Yu J (2012). Cellular glutathione redox homeostasis plays an important role in the brassinosteroid-induced increase in CO_2_ assimilation in Cucumis sativus. New Phytol.

[77] Simkin AJ, Faralli M, Ramamoorthy S, Lawson T (2020). Photosynthesis in non-foliar tissues: implications for yield. Plant J.

[78] Varone L, Ribas-Carbo M, Cardona C, Gallé A, Medrano H, Gratani L, Flexas J (2012). Stomatal and non-stomatal limitations to photosynthesis in seedlings and saplings of Mediterranean species pre-conditioned and aged in nurseries: Different response to water stress. Environ Exp Bot.

[79] Acharya BR, Assmann SM (2009). Hormone interactions in stomatal function. Plant Mol Biol.

[80] Naranjo B, Mignée C, Krieger-Liszkay A, Hornero-Méndez D, Gallardo-Guerrero L, Cejudo FJ, Lindahl M (2016). The chloroplast NADPH thioredoxin reductase C, NTRC, controls non-photochemical quenching of light energy and photosynthetic electron transport in Arabidopsis. Plant Cell Environ.

[81] Kalaji HM, Jajoo A, Oukarroum A, Brestic M, Zivcak M, Samborska IA, Cetner MD, Łukasik I, Goltsev V, Ladle RJ (2016). Chlorophyll a fluorescence as a tool to monitor physiological status of plants under abiotic stress conditions. Acta Physiol Plant.

[82] Maxwell K, Johnson GN (2000). Chlorophyll fluorescence—a practical guide. J Exp Bot.

[83] Liang J-F, An J, Gao J-Q, Zhang X-Y, Song M-H, Yu F-H (2019). Interactive effects of biochar and AMF on plant growth and greenhouse gas emissions from wetland microcosms. Geoderma.

[84] Mathur S, Sharma MP, Jajoo A (2018). Improved photosynthetic efficacy of maize (*Zea mays*) plants with arbuscular mycorrhizal fungi (AMF) under high temperature stress. J Photochem Photobiol B.

[85] Ivanov DA, Bernards MA (2015). Chlorophyll fluorescence imaging as a tool to monitor the progress of a root pathogen in a perennial plant. Planta.

[86] Kreslavski VD, Brestic M, Zharmukhamedov SK, Lyubimov VY, Lankin AV, Jajoo A, Allakhverdiev SI (2017). Mechanisms of inhibitory effects of polycyclic aromatic hydrocarbons in photosynthetic primary processes in pea leaves and thylakoid preparations. Plant Biol.

[87] Zurbriggen MD, Carrillo N, Tognetti VB, Melzer M, Peisker M, Hause B, Hajirezaei M-R (2009). Chloroplast-generated reactive oxygen species play a major role in localized cell death during the non-host interaction between tobacco and Xanthomonas campestris pv. vesicatoria. Plant J.

[88] Abdel Latef AAH, Miransari M: The role of arbuscular mycorrhizal fungi in alleviation of salt stress. In: Use of Microbes for the Alleviation of Soil Stresses. 2014; 2: 23-38.

[89] Jogawat A. Osmolytes and their role in abiotic stress tolerance in plants. In: Aryadeep R, Durgesh T, editors. Molecular Plant Abiotic Stress; 2019. p. 91–104. 10.1002/9781119463665.ch5.

[90] Batista-Silva W, Heinemann B, Rugen N, Nunes-Nesi A, Araújo WL, Braun H-P, Hildebrandt TM (2019). The role of amino acid metabolism during abiotic stress release. Plant Cell Environ.

[91] Johny L, Cahill DM, Adholeya A (2021). AMF enhance secondary metabolite production in ashwagandha, licorice, and marigold in a fungi-host specific manner. Rhizosphere.

[92] Panchal P, Miller AJ, Giri J (2021). Organic acids: versatile stress-response roles in plants. J Exp Bot.

[93] Wang K, Hersh HL, Benning C (2016). SENSITIVE TO FREEZING2 aides in resilience to salt and drought in freezing-sensitive tomato. Plant Physiol.

[94] Zhu J-K (2002). Salt and drought stress signal transduction in plants. Annu Rev Plant Biol.

[95] Yooyongwech S, Samphumphuang T, Tisarum R, Theerawitaya C, Cha-um S (2016). Arbuscular mycorrhizal fungi (AMF) improved water deficit tolerance in two different sweet potato genotypes involves osmotic adjustments via soluble sugar and free proline. Sci Hortic.

[96] Helber N, Wippel K, Sauer N, Schaarschmidt S, Hause B, Requena N (2011). A versatile monosaccharide transporter that operates in the Arbuscular Mycorrhizal Fungus Glomus sp is crucial for the symbiotic relationship with plants. Plant Cell.

[97] BÁRzana G, Aroca R, Ruiz-Lozano JM (2015). Localized and non-localized effects of arbuscular mycorrhizal symbiosis on accumulation of osmolytes and aquaporins and on antioxidant systems in maize plants subjected to total or partial root drying. Plant Cell Environ.

[98] Amtmann A, Armengaud P (2009). Effects of N, P, K and S on metabolism: new knowledge gained from multi-level analysis. Curr Opin Plant Biol.

[99] Wu D, Shen Q, Cai S, Chen Z-H, Dai F, Zhang G (2013). Ionomic responses and correlations between elements and metabolites under salt stress in wild and cultivated barley. Plant Cell Physiol.

[100] Obata T, Fernie AR (2012). The use of metabolomics to dissect plant responses to abiotic stresses. Cell Mol Life Sci.

[101] Hildebrandt TM (2018). Synthesis versus degradation: directions of amino acid metabolism during *Arabidopsis* abiotic stress response. Plant Mol Biol.

[102] Less H, Galili G (2008). Principal transcriptional programs regulating plant amino acid metabolism in response to abiotic stresses. Plant Physiol.

[103] Hauser F, Horie T (2010). A conserved primary salt tolerance mechanism mediated by HKT transporters: a mechanism for sodium exclusion and maintenance of high K^+^/Na^+^ ratio in leaves during salinity stress. Plant Cell Environ.

[104] Garg N, Bhandari P (2016). Silicon nutrition and mycorrhizal inoculations improve growth, nutrient status, K^+^/Na^+^ ratio and yield of *Cicer arietinum* L. genotypes under salinity stress. Plant Growth Regul.

[105] Ma L, Zhang H, Sun L, Jiao Y, Zhang G, Miao C (2012). NADPH oxidase AtrbohD and AtrbohF function in ROS-dependent regulation of Na^+^/K^+^ homeostasis in *Arabidopsis* under salt stress. J Exp Bot.

[106] Chakraborty K, Bhaduri D, Meena HN, Kalariya K (2016). External potassium (K^+^) application improves salinity tolerance by promoting Na^+^-exclusion, K^+^-accumulation and osmotic adjustment in contrasting peanut cultivars. Plant Physiol Biochem.

[107] Mostofa MG, Rahman MM, Ghosh TK, Kabir AH, Abdelrahman M, Rahman Khan MA, Mochida K, Phan Tran L-S (2022). Potassium in plant physiological adaptation to abiotic stresses. Plant Physiol Biochem.

