# Supplementary material for: The multifaceted roles of Arbuscular Mycorrhizal Fungi in peanut responses to salt, drought, and cold stress
Source: BMC Plant Biol. 2023 Jan 16;23:36. doi: 10.1186/s12870-023-04053-w (PMC9841720; doi:10.1186/s12870-023-04053-w)
Supplement: Supplementary file 1 — Additional file 1: Fig. S1. (a) Schematic representation of experimental design and treatments. At 40 days after emergence, both AMF-inoculated and non-inoculated plants were exposed to salt, drought, or cold stress for 7 successive days before measurements and samples were taken. (b) The mycorrhization status of the AMF-inoculated peanut roots (200×). Fig. S2. Effect of AMF on root morphology of peanut plants under stressful conditions. The peanut seeds were inoculated with/without AMF before exposing to normal/salt/drought/cold growth conditions. The roots were excised, washed thoroughly, and scanned on the 8th day after the onset of stress treatments. (a) One representative picture is shown for each treatment. The (b) root volume, (c) total root length, (d) root average diameter, and (e) root surface area were determined. Bars represent the mean values of three biological replicates with standard deviation; asterisks indicate a significant difference in comparison to non-AMF according to Tukey's test (P < 0.05). Fig. S3. Effect of AMF on the accumulations of K+, Na+ and the K+: Na+ ratio of roots (a-c) and leaves (d-f) in peanut plants under stressful conditions. The peanut seeds were inoculated with/without AMF before exposing to normal/salt/drought/cold growth conditions. The root and leaf samples were taken on the 8th day after the onset of stress treatments. Bars represent the mean values of three biological replicates with standard deviation; asterisks indicate a significant difference in comparison to non-AMF according to Tukey's test (P < 0.05). Fig. S4 .OPLS-DA models (a), volcano plots (b), and heat map showing the differential metabolites with MS2 (c) based on the non-target metabolomics in the peanut root samples of “AMF + NaCl vs NaCl”. Fig. S5. OPLS-DA models (a), volcano plots (b), and heat map showing the differential metabolites with MS2 (c) based on the non-target metabolomics in the peanut root samples of “AMF + Drought vs Drought”. Fig. S6. OPL [file 12870_2023_4053_MOESM1_ESM.docx]

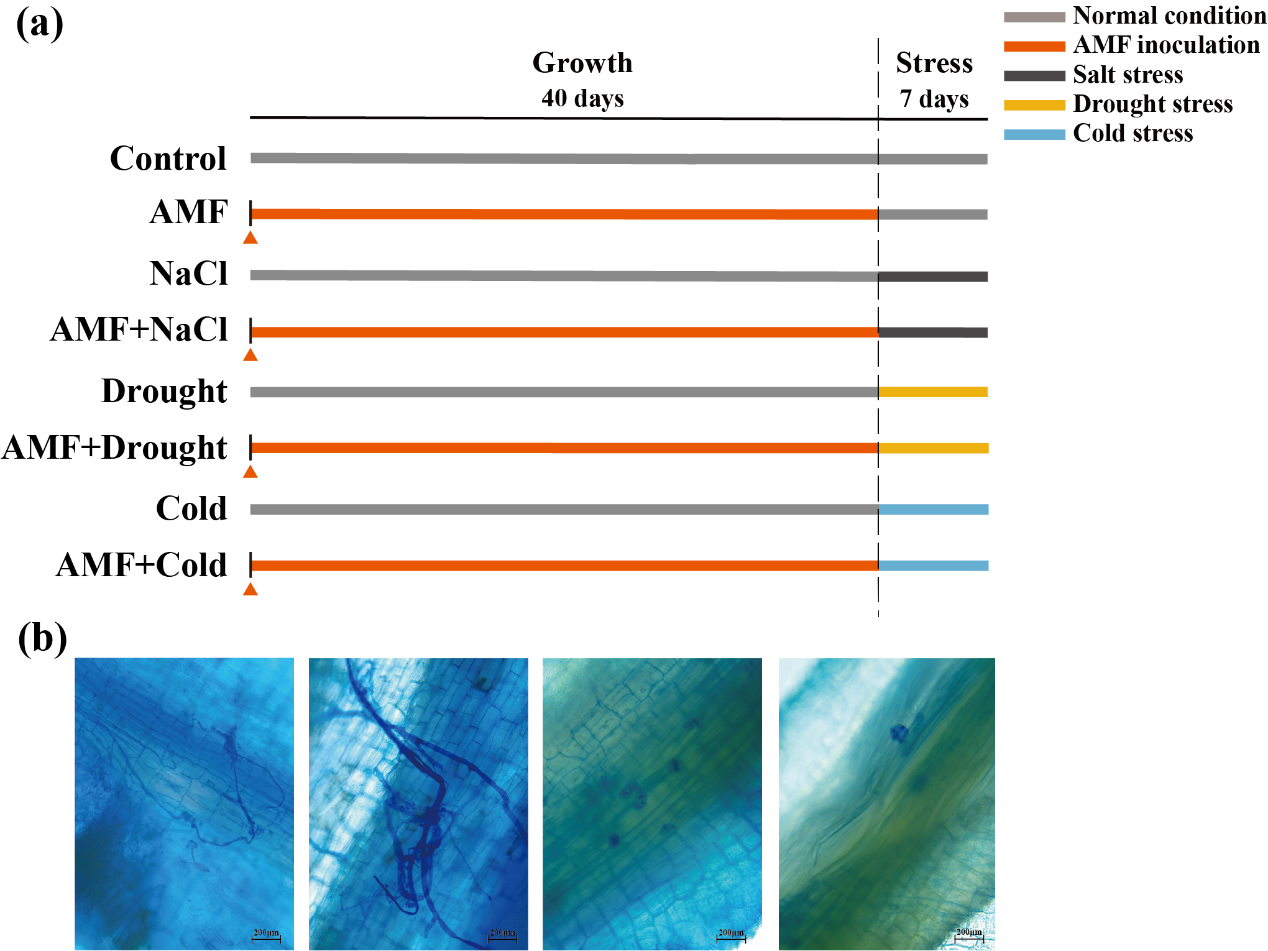


**Fig. S1** (**a**) Schematic representation of experimental design and treatments. At 40 days after emergence, both AMF-inoculated and non-inoculated plants were exposed to salt, drought, or cold stress for 7 successive days before measurements and samples were taken. (**b**) The mycorrhization status of the AMF-inoculated peanut roots (200×).

**
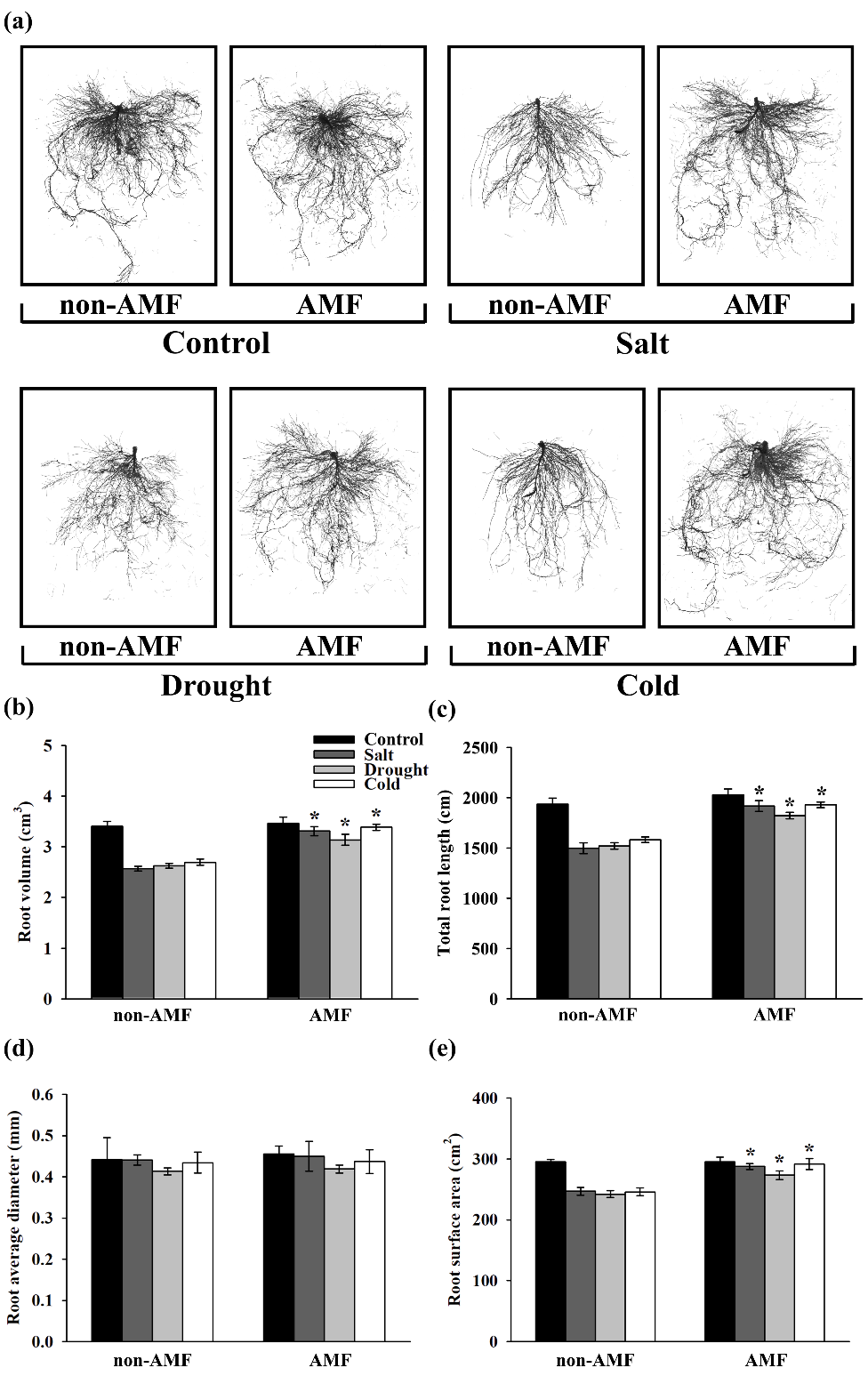
**

**Fig. S2** Effect of AMF on root morphology of peanut plants under stressful conditions. The peanut seeds were inoculated with/without AMF before exposing to normal/salt/drought/cold growth conditions. The roots were excised, washed thoroughly, and scanned on the 8^th^ day after the onset of stress treatments. (**a**) One representative picture is shown for each treatment. The (**b**) root volume, (**c**) total root length, (**d**) root average diameter, and (**e**) root surface area were determined. Bars represent the mean values of three biological replicates with standard deviation; asterisks indicate a significant difference in comparison to non-AMF according to Tukey's test (*P* < 0.05).

**
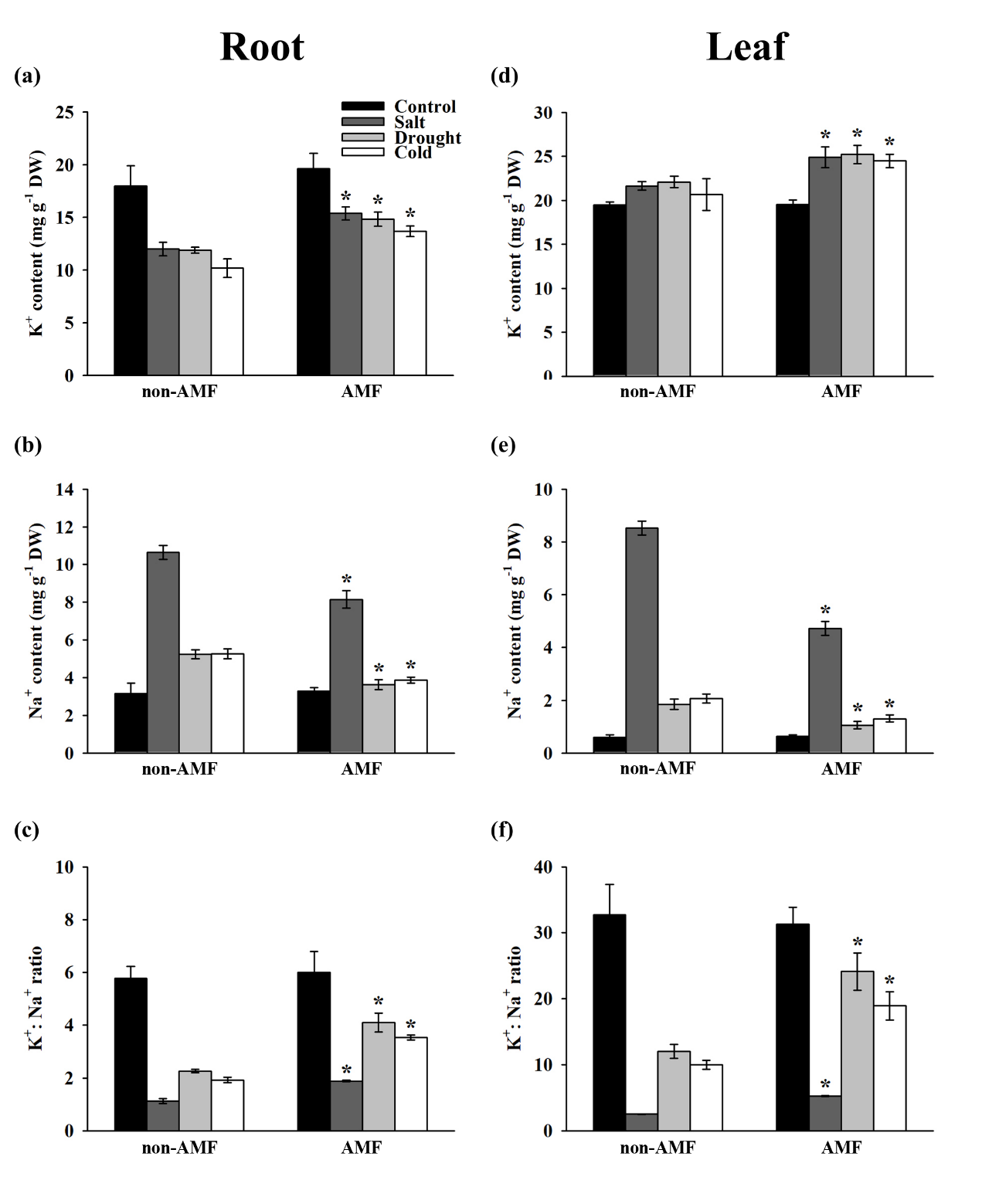
**

**Fig. S3** Effect of AMF on the accumulations of K^+^, Na^+^ and the K^+^: Na^+^ ratio of roots (**a**-**c**) and leaves (**d**-**f**) in peanut plants under stressful conditions. The peanut seeds were inoculated with/without AMF before exposing to normal/salt/drought/cold growth conditions. The root and leaf samples were taken on the 8^th^ day after the onset of stress treatments. Bars represent the mean values of three biological replicates with standard deviation; asterisks indicate a significant difference in comparison to non-AMF according to Tukey's test (*P* < 0.05).

**
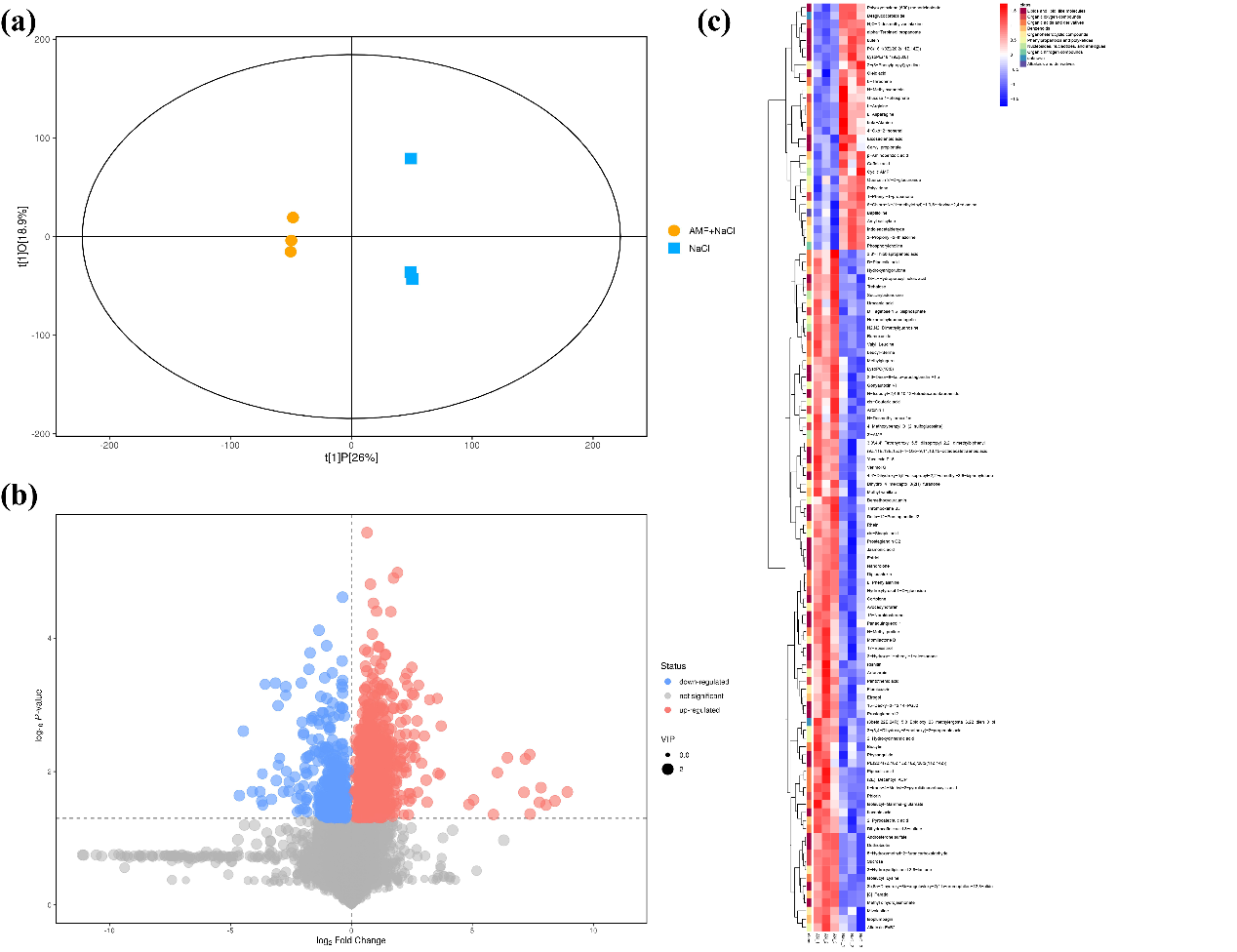
**

**Fig. S4** OPLS-DA models (**a**), volcano plots (**b**), and heat map showing the differential metabolites with MS2 (**c**) based on the non-target metabolomics in the peanut root samples of “AMF + NaCl vs NaCl”.

**
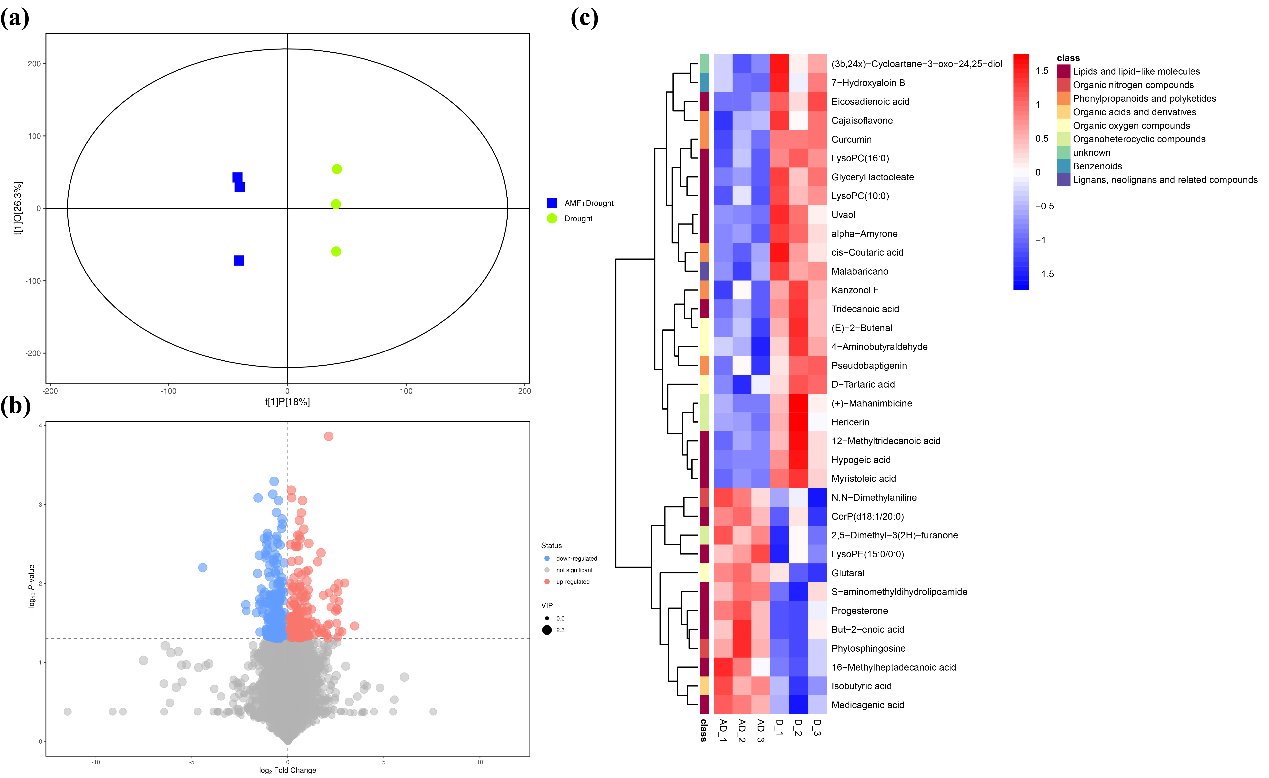
**

**Fig. S5** OPLS-DA models (**a**), volcano plots (**b**), and heat map showing the differential metabolites with MS2 (**c**) based on the non-target metabolomics in the peanut root samples of “AMF + Drought vs Drought”.

**
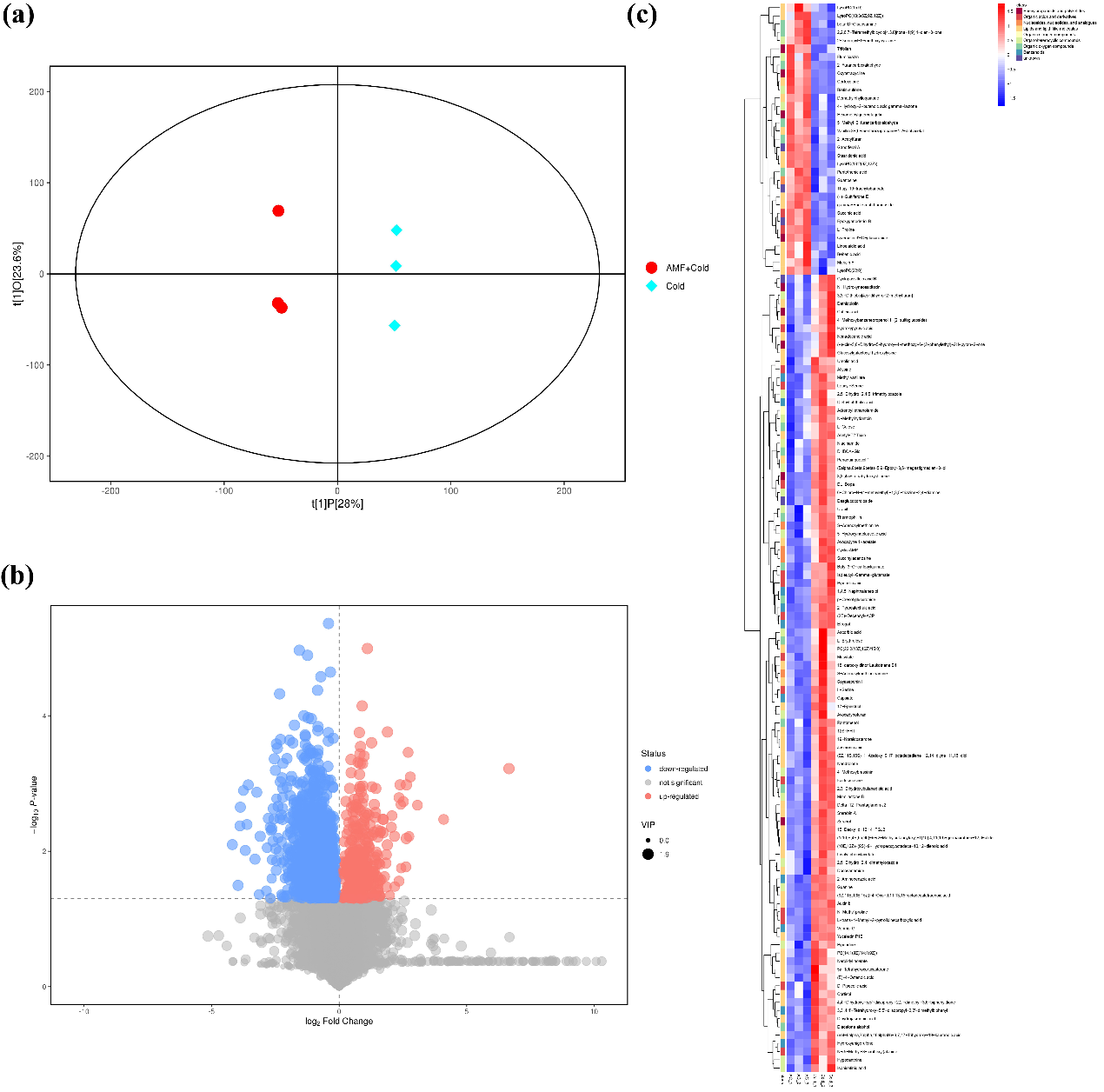
**

**Fig. S6** OPLS-DA models (**a**), volcano plots (**b**), and heat map showing the differential metabolites with MS2 (**c**) based on the non-target metabolomics in the peanut root samples of “AMF + Cold vs Cold”.

**
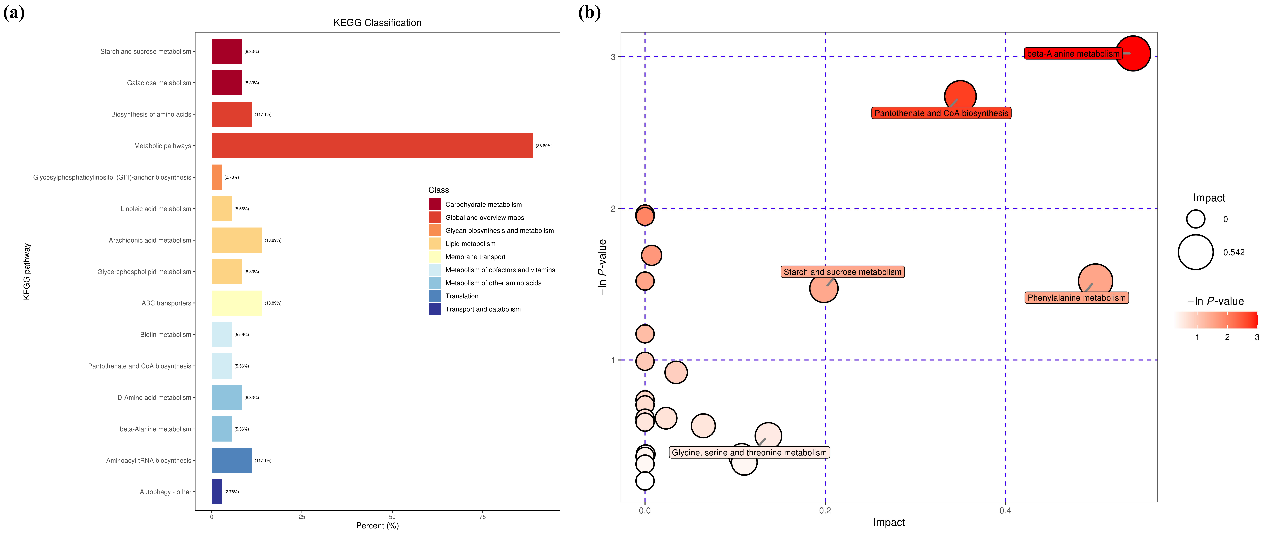
**

**Fig. S7** KEGG classification (**a**) and biosynthetic pathway analysis (**b**) based on the non-target metabolomics in the peanut root samples of “AMF + NaCl vs NaCl”.

**
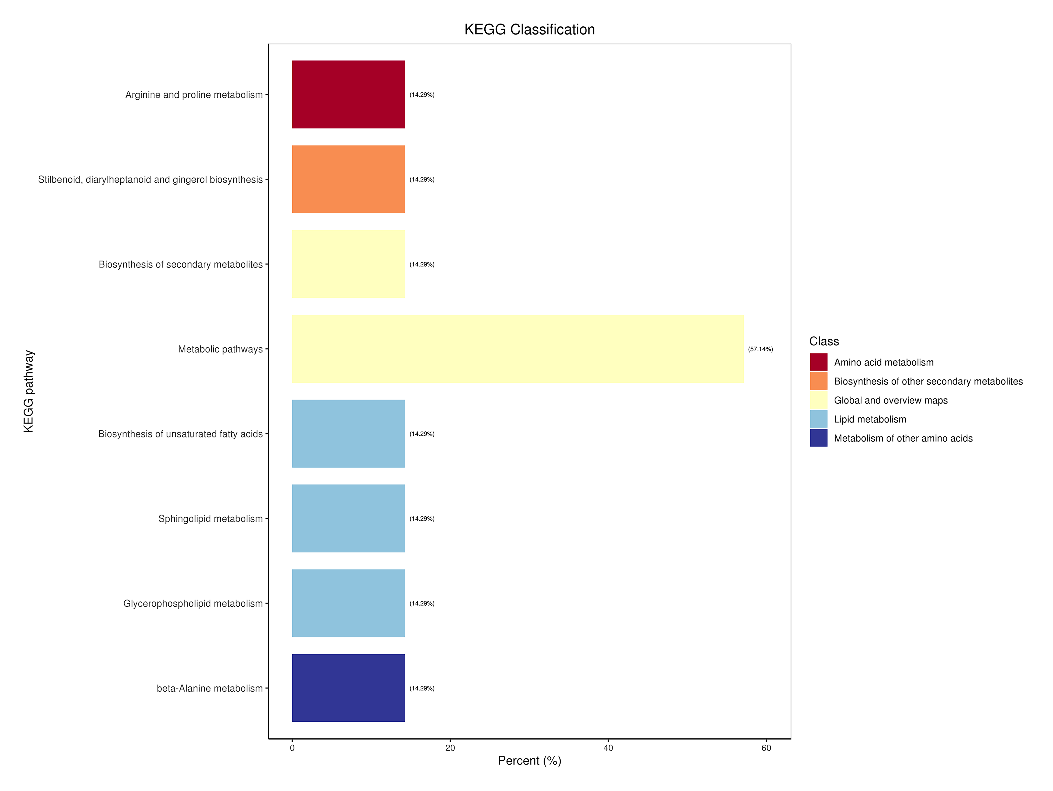
**

**Fig. S8** KEGG classification based on the non-target metabolomics in the peanut root samples of “AMF + Drought vs Drought”.

**
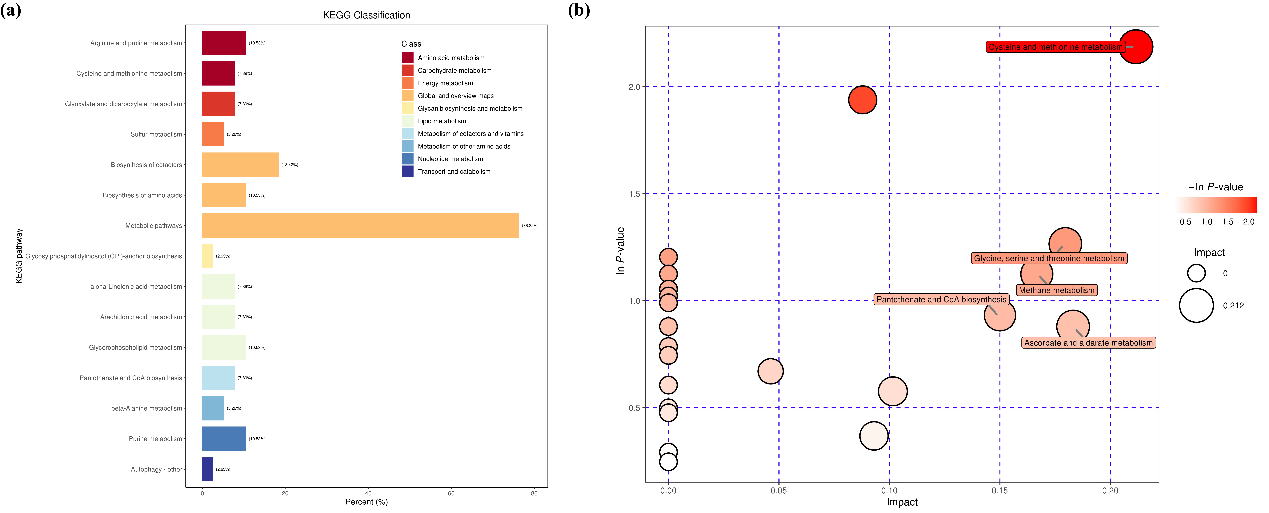
**

**Fig. S9** KEGG classification (**a**) and biosynthetic pathway analysis (**b**) based on the non-target metabolomics in the peanut root samples of “AMF + Cold vs Cold”.
